# A Failure to “Do No Harm” -- India’s Aadhaar biometric ID program and its inability to protect privacy in relation to measures in Europe and the U.S.

**DOI:** 10.1007/s12553-017-0202-6

**Published:** 2017-06-14

**Authors:** Pam Dixon

**Affiliations:** World Privacy Forum, 12625 SW 62ND Ave., Portland, OR 97219 USA

**Keywords:** Privacy, Biometrics, Aadhaar, India, Consent, GDPR, Identity, ID card, Digital identity

## Abstract

It is important that digital biometric identity systems be used by governments with a *Do no Harm* mandate, and the establishment of regulatory, enforcement and restorative frameworks ensuring data protection and privacy needs to transpire prior to the implementation of technological programs and services. However, when, and where large government bureaucracies are involved, the proper planning and execution of public service programs very often result in ungainly outcomes, and are often qualitatively not guaranteeable. Several important factors, such as the strength of the political and legal systems, may affect such cases as the implementation of a national digital identity system. Digital identity policy development, as well as technical deployment of biometric technologies and enrollment processes, may all differ markedly, and could depend in some part at least, on the overall economic development of the country in question, or political jurisdiction, among other factors. This article focuses on the Republic of India’s national digital biometric identity system, the *Aadhaar*, for its development, data protection and privacy policies, and impact. Two additional political jurisdictions, the European Union, and the United States are also situationally analyzed as they may be germane to data protection and privacy policies originated to safeguard biometric identities. Since biometrics are foundational elements in modern digital identity systems, expression of data protection policies that orient and direct how biometrics are to be utilized as unique identifiers are the focus of this analysis. As more of the world’s economies create and elaborate capacities, capabilities and functionalities within their respective digital ambits, it is not enough to simply install suitable digital identity technologies; much, much more - is durably required. For example, both vigorous and descriptive means of data protection should be well situated within any jurisdictionally relevant deployment area, *prior to* in-field deployment of digital identity technologies. Toxic mixes of knowledge insufficiencies, institutional naïveté, political tomfoolery, cloddish logical constructs, and bureaucratic expediency must never overrun fundamental protections for human autonomy, civil liberties, data protection, and privacy.

## Introduction

In recent years, governments have acted to build pervasive digital identity ecosystems.^1^ Such actions represent the desire by world societies to advance beyond their inefficient paper-based existence, to highly integrated and interoperable digital economies, where at least a form of digital identity has been determined to be essential to such transforms [2]. The installations of such systems, which often include biometric data components, are technical undertakings that intertwine and network data linkages - sometimes across multiple political or economic jurisdictions. In cases of such deployments, networked digital identity systems can correspondingly pass - a single identity for example, across vastly diverse online and offline health, finance, education, and government data systems. The rewards associated with the implementation of such digital identity systems may include greater population access to public and, or commercial services. However, there is also the prospect for the presence of substantial long-term risks in relation to the utilization of digital identity systems; such risks must therefore be addressed, and without fail.

In a widespread distribution of networked digital identity systems, ‘an identity or ID,’ can become pervasive and persistent, as that ID is deliberately conveyed to, and made resident within many connected systems, and can therefore, be used as a potent mechanism of social or political control, or personal surveillance, as a biometric identifier that can uniquely identify an individual and his movements among multiple systems. Historically, pervasive and persistent identity systems have presented risks to individuals, even when identity documents have been in paper forms. A sobering historic case embroils the Republic of Rwanda, where personal identity documents that included ethnicity, were used to aid, and to expedite, genocidal activities [3].^2^

Digital forms of identity systems, when fully developed and deployed, are expected to be more powerful and efficient tools of identification than legacy paper systems. The power and efficiency proffered by such tools, both pose and mount a great urgency to identify, and to mitigate modern risks associated with system breach and the compromise of vital information in those identity systems, and to ensure that digital identity systems do not become tools of suppression, oppression, exclusion, or discrimination.

Of the digital biometric identity systems in existence today, the most notable information exploitation case is that of the Republic of India. India’s biometric identity system, called the *Aadhaar*,^3^ has more than one billion enrollees, yet remarkably, the Indian government failed to legislate much needed comprehensive data protection and privacy laws, even though the legislative process had once well advanced,^4^ and legislative language sits in waiting.^5^ The *Aadhaar* system, having been deployed rapidly, is less than a decade old and its history, development, and impact has been well documented. As such, India’s *Aadhaar* provides a unique and prominent case study for how risks that are endemic to identity systems have developed, and have since been welded-in to their digital biometric system. To fully understand the *Aadhaar* policies as proposed, then enacted; and to comprehend how the existing policies have failed, it is essential to first be properly introduced to how biometrics function in digital identity systems.

### The role of biometrics in digital identity systems

Biometrics are at the center of an emerging set of modern policies related to determining one’s identity, and establishing one’s identity is key to achieving any number of policy goals, from catching criminals, to establishing efficiencies within the health care sector, to providing an identity deemed trustworthy enough for opening a bank account. Biometrics is essentially the authentication or identification of an individual based on personal or behavioral characteristics [6]. A fingerprint is probably the best-known biometric; fingerprints have been used in ink-and-paper forms for law enforcement purposes for decades, for example, the US government began maintaining a database of fingerprints in 1904.^6^ The US Federal Bureau of Investigation’s Integrated Automated Fingerprint Identification System (IAFIS) database was an early (1999) iteration of a digitized fingerprint database,^7^ which allowed digitized fingerprints to be exchanged among law enforcement agencies. Most recently, the FBI has incorporated additional biometrics, such as iris and facial recognition, in an updated system called Next Generation Identification (NGI), which was launched in 2011 and is now in its fourth increment.^8^ Europe has similar databases that have undergone comparable paper-to-digital transformation.^9^

In health care settings, it is becoming increasingly common for healthcare providers to request that patients and healthcare workers provide a palm print, a fingerprint, or another biometric for unique identification.^10^ For patients, a biometric can serve to identify the patient and disambiguate similarly named patients from each other. For health care workers, complexities around using passwords to unlock digital records have increasingly failed, and biometric use is meant to replace password use. For example, passwords for sensitive health databases have been written on note pads and placed under keyboards as memory devices, and have been a well-known risk in hospitals.^11^ With biometric identification, the workers’ fingerprint or palm print can unlock a health records system, much like some types of mobile phones can be unlocked with a fingerprint or a facial biometric.^12^ The health care arena is not alone in the struggle regarding passwords, and biometrics is being seen as a way to broadly address these challenges.^13^

As discussed, the practice of collecting biometric information such as fingerprints from people is not new, and neither is the use of biometrics for identification or authentication.^14^ There is an important distinction to be made, however, between individual and local use of a biometric identifier, versus the use of biometric identifiers as part of a true digital identity ecosystem. For example, using a biometric such as a fingerprint to unlock a mobile phone, or in the case where a single bank or a health care provider creates its own database of customer biometric information -- these are localized, non-networked uses of biometrics. They are essentially silos of biometric information.

What is new, however, is the way digital identities enhanced with biometrics are being widely linked, sometimes across all sectors and sometimes nation-wide, to create powerful *ecosystems* of identity information.^15^ Digital identity, when in an ecosystem, is not just about having a biometric used locally on a mobile phone. A digital identity ecosystem involves a complex network in uses of identity that ranges from multiple government uses to commercial uses of identity as a service.^16^ Joseph Atick describes digital identity ecosystems as “a platform consisting of a collection of technologies, processes and policies that are integrated together to enable unique natural persons to prove, unambiguously and securely, who they are to an information system and to empower them to assert their legal rights in a digital context.” [2] The ability to merge inexpensive computer storage, and substantial computing power has increased both the capacity for building these large identity systems to accommodate digitized biometric elements from populations, and the appetite for doing so.^17^

One of the driving factors toward adoption of biometric identity systems at this time is the reduction in error rates. New research and development in neural networks, and deep machine learning, are improving the existence of persistently high error rates in the use of biometrics, rates which had previously served as a substantial disincentive to the deployment of biometrics systems to resolve numerous policy matters, such as wide-scale use for government subsidy disbursements.^18^ However, as systems improve and error rates decrease, a significant set of objections to biometrics is increasingly diminished as a point of contention. This will have an impact on policy decisions regarding how, where, and when digital identities will be used, and will almost certainly lead to greater spread, and use of biometric identification and authentication.^19^ With greater information technology automation, lower component costs, and increased accuracy in results, the use of biometrics is poised to enter conventional service, away from the small enclaves of expert user communities and toward a broader distribution of much larger, and less expert populations.

### The impacts of digital biometric ecosystems

Large digital identity ecosystems come with increased efficiencies, and they also come with increased risks. Biometrically enhanced identity information, combined with demographic data such as address, age and gender, among other data, when used in increasingly large, automated systems creates profound changes in societies, particularly in regards to data protection, privacy, and security. One of the most significant changes is the precipitous decline of *privacy by obscurity,* 20 which is essentially a form of privacy afforded to individuals inadvertently by the inefficiencies of paper and other legacy recordkeeping. Now that paper records worldwide are giving way to more efficient digital record-keeping and identification, this form of privacy is being extinguished, and sometimes without commensurate data privacy protections put in place to remedy the effects of the changes. One can compare the clumsiness of legacy paper-and-ink fingerprint cards held by local institutions, to the efficiencies of modern digital multi-modal biometrics databases,^21^ which may include millions of digital fingerprint templates, combined with other types of biometrics, all of which can be searched rapidly from a single computer terminal. Similar changes can be seen in digitization of health records, whether or not a biometric is included [17]. Digitization serves to create a rich and deep pool of information, often instantly accessible, and introducing consequently, profound changes into how identity information works.

These changes create great responsibility for policymakers to ensure the responsible use and interpretation of identity and biometric data. Broader deployment and adoption also increases the importance of providing safeguards – procedural, substantive and restorative – to diminish or respond to potential deleterious side effects of biometrics. The use of digital identity systems and biometrics for identifying or authenticating individuals need not be onerous, if appropriate protections are in place. However, if appropriate protections are not in place, the use of large-scale biometric identity systems can also be used for purposes of social control, surveillance, and repression. Therefore, adequate protections are of utmost importance to guide and direct digital biometric identity systems.

But what protections are in place now - for the emerging impacts from the utilization of large-scale information system housing digital biometric identities? How do existing manners of protections differ from each other, and why do the differences between existing measures to protect matter? A required scholarly inquiry in light of the rise of populism and extremist views worldwide should more specifically be, to assess whether those protections that exist, extend sufficient protections for populations by ensuring that persons are able to conclusively express their Consent to the collection, and the use of their personal information. The basis for research, and the following expression of the research findings in this article began in 2010, where the author’s travel to the Republic of India intersected with the launch of their national identity card for each of the nation’s billion citizens, incorporating biometric values.

The impact of witnessing such a profound and rapid shift in the use of information technology for building an identity ecosystem, where *privacy by obscurity* 22 went from being in abundant force, that is, an abundance of paper records with limited access, to being a receding memory in a mere few calendar years, countrywide, still resounds today. Men and women living in remote villages, some without plumbing in their homes and many living in extreme poverty without access to modern technology, in the space of a few years underwent sophisticated biometric enrollments and began using their biometric identity for access to government subsidies such as rations. Women, who used to take inches-thick paper booklets holding generations of their families’ health care history written carefully in script, now access health care through their *Aadhaar* identity with a digital authentication, for example, through a fingerprint scanner or a mobile phone.^23^

Are these changes all positive? Alternatively, are they problematic? To date, biometric deployment in India gives an extraordinary and rare view into some of the most challenging policy issues associated with swift, large-scale digital identity and biometric deployments; absent any connected regulatory and policy guidance. India’s *“Aadhaar”* system,^24^ a biometric national identity system with a centralized database, has the stated goals of delivering services, reducing fraud and increasing efficiencies. But the *Aadhaar* system also represents a near-end state of a large-scale digital biometric identity system deployed during its formative years without direct legislative privacy, or ethics constraints.

As the *Aadhaar* began deployment, there was no legal framework set forth to guide the implementation or use of the card. Even now, comprehensive data protection and privacy legislation guiding how the *Aadhaar* can be used has not been passed.

Of particular concern is the profound mission creep associated with the *“Aadhaar”* digital system. Initially the *Aadhaar* was only used for subsidies, now it is used for bank accounts, medical records, pension payments, and a seemingly ever-growing list of activities. While it was launched as *‘voluntary,’* and for limited purposes, *Aadhaar* enrollment is now *‘mandatory’* and must be present to receive many national government, and Indian State benefits and services. Additionally, *Aadhaar* enrollment has become both functionally and practically mandatory even beyond those levels.

The Republic of India is not alone in its deployment of biometric authentication within the course of identity management. Biometrics is a near-global technology concern, and it has become important to closely study the policies deployed in India, as well as other political jurisdictions, to determine the negatives and the positives in deployment and impact. Such studies are important, as there is little to no expectation of a reversal in the use of biometrics within identity management.

Now that biometrics technology and processes are increasingly dispersed globally, it is equally as important for evaluations of policy impacts across legal jurisdictions to be undertaken. Scholarly evaluation of policy constructs for digital biometrics systems comprises an under-researched area. A variety of biometric systems have undergone significant *technical* evaluations conducted by a variety of experts, for example, the US National Institute of Technology and Standards (NIST) has conducted the Face Recognition Grand Challenge, the Iris Challenge Evaluation, and others.^25^ These evaluations have been important in determining the accuracy and efficacy of differing biometric systems. Nevertheless, what has been missing is a concomitant *policy* evaluation of biometric digital identity systems.

## India’s national digital biometric ID system and policies

India, to date, has implemented a systemic digital biometric identity system.^26^ The system, called *Aadhaar*, or *Universal ID* (UID), is persistent and pervasive, and it is used across sectors such as banking, health, and government. A significant majority of India’s residents now have the *Aadhaar* ID; as of 2016, 97% of adult Indians, and 67% of children are enrolled.^27^ In 2010, the first enrollees were given iris scans and registered in the then-voluntary *Aadhaar* system for the stated purpose of granting them easier access to subsidies from the government.

By 2016, the *Aadhaar* system reached and then surpassed one billion enrollees. Despite the near-ubiquity of the *Aadhaar*, and its increasing use in everyday life, India’s government has still not passed national data protection and privacy legislation for the *Aadhaar* identity system, even though suitable proposals have been drafted that would provide a version of globally accepted and widely implemented data protection standards.^28^ That the government of India has repeatedly bungled providing important data and privacy protections for its people is disquieting. Milan Vaishnav, in his book on modern Indian politics, writes:

*Unlike many countries in the West, India embarked on its democratic journey without first possessing capable institutions of governance. Whereas many advanced industrialized democracies built strong states over centuries before embarking on a process of political liberalization, India instituted universal franchise from the outset, operating under the constraints of a relatively weak institutional framework. Over time, as the stresses of political, economic, and social change have grown, the country's institutional framework has proven too frail to cope.* 29

Vaishan’s description of India’s institutional framework as conferring universal franchise too soon, and as ultimately “too frail to cope” is an apt description of both why the *Aadhaar* system appealed to India, and why the legislative protections for the *Aadhaar* system have been routinely deferred. The resulting lack of data privacy legislation in India has been consequential; in 2016, the *Aadhaar* was made mandatory,^30^ but still without accompanying privacy legislation. As a result, the uses of the *Aadhaar* have expanded sizably, growing from a narrow subsidy program to one that includes banking, health, scholarships, and numerous public services.^31^

To understand the consequences of India’s decisions to not provide adequate data protection in the *Aadhaar* system, it is fitting to be familiar policies and activities in more detail.

### The *Aadhaar* identity system

India’s biometric identity program, *Aadhaar*, issues a 12-digit^32^ unique identification number to enrollees. The Unique Identification Authority of India (UIDAI) is the regulatory authority that issues the *Aadhaar* number,^33^ and it retains the cardholders’ demographic and biometric information^34^— including iris scans — in a national, centralized database called the Central Identities Repository.^35^ The biometric data associated with the demographic data is meant to ensure the proper demographic information, like gender, is matched to the proper person; it is also meant to ensure that transactions based on the *Aadhaar* system are non-duplicative, and can be effectuated from any location in India, through online or other electronic means.

*Aadhaar* holders can use mobile devices, combined with a Personal Identification Number or PIN, or can use a biometric, via a biometric reader or kiosk to validate their identity. The *Aadhaar* central database can be accessed by a variety of individuals and entities, ranging from employers, to banks, to law enforcement - in real time, or near-real time.^36^

Centralized identity databases, however, have been controversial, because of the inherent security risks and policy frailties that have come to be associated with them.^37^ Regarding security risks, data breach - either purposeful breach from unauthorized access, or inadvertent leakages due to technical or clerical errors, are persistent threats. Thus far, there is reasonable proof that the *Aadhaar* system has already had some security leakages that could be deemed to be of the inadvertent variety. In early 2017, a spate of articles were published about the ease of locating Excel files that had been posted online erroneously, originating from various Indian government offices, replete with *Aadhaar* numbers and demographic data, retrievable through a simple Google search; one breach resulting from a programming error led to the publication of the bank details of a million *Aadhaar* pension beneficiaries on a government website.^38^ One journalist found the details of several thousand enrollees, including their *Aadhaar* numbers, posted online by a handful of Indian government websites [22].

Regarding policy risks, India’s *Aadhaar* system has exhibited significant weakness regarding the lack of attention to policy, including policies regarding basic data protection and privacy practices. The government of India has bungled a series of opportunities to enact data protection and privacy legislation for the *Aadhaar* system;

### *Aadhaar* policy in India

When the UIDAI began enrollments for *Aadhaar* in 2010,^39^ there was no law in place relating to the *Aadhaar* biometric program, nor any privacy provisions for the biometric data it was to collect. The National Identification Authority of India Bill 2010 was introduced two months after enrollment began in order to address privacy issues in the *Aadhaar* system [23]. However, the Parliamentary Standing Committee on Finance rejected the 2010 bill. This was India’s best opportunity to pass early legislation before the mass enrollment of its citizens in the *Aadhaar* system. The Privacy Bill of 2011 was put forward again in 2012 to attempt to provide data protection for the *Aadhaar* system, but it was not passed.^40^ It is difficult to understand why India did not act to put data protection legislation in place in the early years of *Aadhaar*. Attorney and legal scholar Usha Ramanathan has characterized the reasons for the early bills’ rejection as being in part the disorganization that surrounded the early phases of the project:

*…That the project had carried on despite a bill pending in parliament; that ‘illegal’ immigrants too were being enrolled; that there was no clarity of purpose; that the NPR [National Population Register] and the UID remained unreconciled; that the collection of biometrics had not been debated in parliament and the Citizenship Act and Rules had not been amended to permit such collection; that biometrics is expected to fail to the extent of 15 percent because of “a large chunk of the population being dependent on manual labour”; that the Ministry of Home Affairs had raised serious security concerns; that there were apprehensions that what was claimed to be voluntary could become a case of denial of even food entitlements if they do not have an Aadhaar number; that linking Aadhaar to entitlements would not solve the problem of correct identification of beneficiaries; that experience and analysis of the project in the UK had not been drawn upon.* [24]

In the year 2012, enrollment in the *Aadhaar* program continued, despite the lack of policy protections. Led by Justice A.P. Shah, a *Group of Experts* from India formally met in 2012 to consider and investigate applicable international privacy standards for India. In October 2012, the Group submitted a report to the Indian government recognizing principles of privacy protection.^41^ The report was sophisticated in its delineation of the privacy implications of *Aadhaar*, and contained nine principles. These principles closely resembled the Organization for Economic Cooperation and Development’s (OECD) Fair Information Practices (FIPs),^42^ and yet the principles had been thoroughly adapted for Indian culture. The report was, and still is, a cornerstone to privacy thought in India.^43^ The report, 91 pages in length, is the first major articulation of Indian thought regarding modern privacy. Justice A.P. Shah stated: “These principles, drawn from best practices internationally, and adapted suitably to an Indian context, are intended to provide the baseline level of privacy protection to all individual data subjects.”

A group of reformers wrote a new bill that incorporated the recommendations of the *Group of Experts*. The result was the Privacy Bill of 2014. It proposed the establishment of a Data Protection Authority as a regulatory body, with enforcement powers over Privacy violations associated with the misuse of biometrics, among other protections that the *Group of Experts* had outlined in its 2012 report. However, the 2014 bill has languished; it still has not yet been officially tabled in Parliament.^44^ *The Group of Experts*’ report and the Privacy Bill of 2014 remain the clearest vehicles that would create a potential path forward for India regarding data protection legislation of its *Aadhaar* identity system.

While the bills were being drafted, the *Aadhaar* project expanded its mission to include certain other activities, for example, the receipt of certain types of government subsidies by individuals. Formal complaints to India’s High Court followed, and soon a parallel series of policy developments occurred. First, the High Court of India made a series of decisions regarding ‘voluntariness’ associated with the *Aadhaar* system, ultimately issuing an interim order in 2015 that the *Aadhaar* card was not to be mandatory, and residents could not be forced to enroll. The judges restricted mandatory use of the *Aadhaar* card to the Liquid Petroleum Gas (LPG) subsidy^45^ and certain other government benefits, and instructed that an education campaign be carried out to make residents aware of biometrics, and the *voluntariness* 46 of the card.^47^

Even though the interim High Court ruling regarding *voluntariness* was in place, in March 2016 the government nonetheless proposed *The Aadhaar Act,* the (Targeted Delivery of Financial and Other Subsidies, Benefits and Services).^48^ The bill proposed, and allowed for, expanded uses for the *Aadhaar* program, and the bill made some uses of *Aadhaar* mandatory. The Bill was passed as a “money bill,” instead of being debated by a Parliamentary panel. What this meant in practice is that the bill essentially passed as a part of a much larger budget act, without its own dedicated debate and vote.

Many objected to the way the *Aadhaar* Bill was passed.^49^ The *Aadhaar Act* is now the current statutory backing for the Aadhaar identification system.^50^ The Act was updated in September, 2016 with regulations, which expanded the power of the Unique Identification Authority of India and gave the government of India substantial ability to access the *Aadhaar* data, with broad abilities to use the data for law enforcement purposes.^51^ Biometric data in *The Aadhaar Act* is defined as: “*biometric information” means photograph, finger print, Iris scan, or such other biological attributes of an individual as may be specified by regulations*.”^52^ Should the government of India decide in the future to begin linking DNA information to the *Aadhaar* system under *The Aadhaar Act*, the language of this definition would allow for it under the phrase “other biological attributes.” Given the broad access of the government to the Aadhaar database, including for law enforcement purposes, and the ability of the Indian government to link DNA data to the card at a future data, combined with the lack of privacy protections in *The Aadhaar Act*, it is regrettable that the National Identification Authority of India Bill 2010 -- which contained privacy provisions -- was quietly withdrawn from Parliament after the passage of *The Aadhaar Act*, thus making it even more difficult for the Indian government to debate and pass a privacy bill for *Aadhaar*.

*The Aadhaar Act* as passed is not a comprehensive privacy bill for the *Aadhaar* system. *The Aadhaar Act* contains procedural directives, including a section on some aspects of information security. *The Aadhaar Act* does not implement privacy, nor full data protections as embodied in the Privacy Bill of 2014 and the *Group of Experts’* report, and as such, the *Aadhaar Act* should not be construed as a “privacy law.”^53^ As mentioned, the *Aadhaar Act* allows for expansive use of the identity system by the government, including for national security purposes, and potentially external entities, subject to the Act’s regulation.^54^ The *Aadhaar Act* does not even give protections up to the level of the *Principles on Identification*, a joint policy document of the World Bank Group, the United Nations Development Programme (UNDP), and other signatories describing principles of privacy and non-discrimination, among other principles.^55^

Since the *Aadhaar Act* became law in March 2016, rapid mission creep for *Aadhaar* use has ensued. Now, individuals must have an *Aadhaar* number to file taxes,^56^ apply for and receive school scholarships,^57^ to book rail tickets,^58^ for religious worship in some private temples,^59^ and for public-sector jobs such as teaching and public health positions. Other uses, such as linking the *Aadhaar* with banking records, health records, and with insurance company programs^60^ are not described as mandatory, but are strongly encouraged. It is becoming increasingly difficult to conduct routine tasks in India without an *Aadhaar* card. Not surprisingly, there is now a system in place to register newborns in the *Aadhaar* system directly in hospitals.^61^ Additional activities that now require *Aadhaar* enrollment include: children’s midday meal,^62^ training and medical appliances for disabled persons, [26] and anti-retroviral therapy for people with HIV, among others, including rehabilitation to women and others attempting to be rescued from prostitution.^63^

Although absent dedicated data protection legislation for the *Aadhaar* system, India has some existing privacy laws. These can be found in the Information Technology Act of 2000, which was amended in 2008. Subsequent to the 2008 amendment, the Indian government issued four additional Rules for the Information Technology Act, known presently as the *“2011 Rules.”* [29] These rules incorporate the idea of Sensitive Personal Data or Information, particularly under section 43A of the Act. The laws and regulations of many nations treat a class of data as “sensitive,” and attach greater protections to the collection, storage, usage and sharing of those types of data. A formal clarification from the Indian government states that the 2011 Rules are regarding sensitive personal data or information and are applicable to “any person located in India.”^64^ Sensitive data is discussed in Section 3 of the Act, and include: passwords, financial information such as bank account, credit or debit card or other payment card information, physical, physiological and mental health condition, sexual orientation, medical records and history, and biometric information.^65^ This data may only be shared with Consent:

*…Any such disclosure of sensitive personal data or information by body corporate to any third party shall require prior permission from the provider of such information.* 66

The idea of *‘Consent’* is not clearly presented in law, other than the following statement that, “*Consent includes Consent given by any electronic mode of communication*.”.^67^

As a reminder, the Information Technology Act is not a full vehicle for robust implementation of the aspirational goals embodied in the *Group of Experts’* 68 nine principles, nor the Fair Information Practices. While it accomplishes some goals, privacy and data protection are an add-on, not a focus. The sensitive data and Consent policies, while welcome, deserve their own legislative vehicle.

Praise has been given to the *Aadhaar* system for its financial inclusion of the poor^69^ and reduced benefits “leakage.” [32] While increased inclusion is a positive development, there are significant concomitant problems regarding exclusion. On a technical level, the State of Jharkhand has a 49% failure to match rate, and Rajasthan has a 37% failure to match rate, according to the Indian government.^70^ The “failure to match” rate of 49% in Jharkhand means that 49% of *Aadhaar* holders in that state cannot be matched to their digital biometric identifier. Individuals who fail to match do not get their benefits, which creates exclusion based on fail to match errors.^71^ These non-match rates and exclusion errors are significant figures, and cannot be ignored. What happens to people who cannot check in for work? What happens to people -- including children -- who fail to match and do not get their food or fuel? These are acute concerns. There are additional substantive disagreements that the *Aadhaar* has been beneficial; for example, beyond the exclusion errors, there is discussion that the way the *Aadhaar* data are stored has the “potential to perpetuate caste identities” [33].

The mere size and perfusion of the *Aadhaar* technology does not mean that the inaction of the Indian government to pass comprehensive data protection and privacy legislation for the Aadhaar system has gone unnoticed, or unchecked. Data protection and privacy weaknesses in the *Aadhaar* system are beginning to garner more notice outside of India [34]. The judicial branch of India’s government, for its part, has issued decisions that contrast with the government’s inattention to legislating data and privacy protections for *Aadhaar*. Complaints about the *Aadhaar* system made to India’s High Court focused on privacy *voluntariness*.^72^ The Court found in favor of these complaints and stipulated that the production of an *Aadhaar* card would not be a condition of receiving benefits. (The issue of privacy was set aside for a later hearing.) The Court reaffirmed its decision regarding *voluntariness* 73 again in 2016.^74^ Thus far, despite the Court’s declarations, the rapid enrollment of *Aadhaar* without data privacy regulation has continued unabated. And despite the Court’s declarations, *Aadhaar* is no longer fully voluntary. Much depends on how the government of India decides to address both the discrepancies of its actions in light of the High Court decision, and its deficits regarding data protection and privacy legislation for the *Aadhaar* system.

India is in a difficult position. It has developed an extensive digital biometric ID that is being used in ever-increasing situations, and at the same time the *Aadhaar* is being increasingly criticized for facilitating exclusion and other problems, including for vulnerable populations. Still the government of India has not passed data protection legislation, despite having draft legislation available to consider. Additionally, the existing authority allowing the *Aadhaar* digital identity system to exist, also grants the Indian government expansive powers to access the *Aadhaar* database.^75^ Unless and until India’s proposed bill, The Privacy Act of 2014, or similar legislation is passed, its privacy protections simply do not rise to the level of the baseline standards set forth by the *Group of Experts* 76 in 2012, which were generally based on the internationally accepted Fair Information Practices.

Issues of Consent, secondary usage, health privacy protections around biometric linkages, and mission creep have become prominent challenges in the *Aadhaar* digital identity system. With its insufficient legislative protections, or even any self-regulatory constraints, the reputation of the *Aadhaar* digital identity system is at risk, as is the autonomy of *Aadhaar* users. India is having increasing difficulty reconciling its lack of data protection policy with its own citizens, who are speaking up in increasing numbers about problems with Aadhaar.^77^ Even its legislators are protesting; Sitaram Yechury, CPI(M) General Secretary and Rajya Sabha Member, has called *Aadhaar* “a database for a totalitarian state.”^78^ Ultimately, India will also have difficulty with other economic jurisdictions that have formal data protection regulation in place. Of these jurisdictions, Europe is a particularly important consideration due to its robust data protection regulations.

## Europe’s general data protection regulation and biometrics

The European Union (EU), long a driving force in advancing privacy protections and forcing adherence to those standards extra-territorially,^79^ is in the midst of the implementation period of the first significant revision of its consumer privacy and data protection laws in the last quarter century. By late May of 2018, anyone doing business within the EU’s 28 member nations will need to abide by new mandates and limitations imposed by the *General Data Protection Regulation* (GDPR).^80^ Regarding European policy with respect to both privacy and biometrics use, it is crucial to understand the broader global implications of GDPR’s implementation.

The EU, through the new data protection regulations articulated in the GDPR, has sought to exercise greater control over data protection and privacy matters than the existing Data Protection Directive, EU 95/46.^81^ Increased protections for use of biometrics are a part of this. The GDPR is a complex and lengthy regulation that incorporates a sophisticated, comprehensive approach to privacy, civil liberties, and incorporates the use of new technology deployments that have the potential to impact human autonomy. The wager that Europeans made, was that trade with the EU is so consequential that most other legal jurisdictions and corporations the world over - who want access to the EU market and its 550 million plus residents’ data – would agree to comply with newly established European policy. The essential trade functions, therefore, have become a means for changing behavior worldwide, and this is true for biometrics, as it is to be for other aspects of privacy as well.

Countries and economic or political jurisdictions outside of the EU that permit the widespread use of biometrics in their respective societies will either have to have in force regulations that meet EU standards, or they will need to have a dual system that imposes definitive protections and standards for the biometric data of EU residents, and then a separate system implementing the standards of their own jurisdiction. This will be true, for example, in the case of the Republic of India. As discussed earlier in this article, India has not passed comprehensive data protection legislation for its digital biometric identity system, *Aadhaar*. In the case of the United States, the *EU-US Privacy Shield and Swiss-US Privacy Shield* 82 agreements are the primary instruments that will oversee and consider all matters related to privacy considerations between the EU and US jurisdictions.^83^

Within its own jurisdiction, European data protection policy is far-reaching and inclusive. The data protection regulation includes data protection and privacy regulation across sectors, applying broadly to data uses in the entire jurisdiction. Banking, health, and education interests, for example, do not have separate privacy regulations as can be the case in other economic jurisdictions, as is the case for example, in the United States. Additionally, European policy is complex in part because of the pluralistic nature of the EU, and competition between, and amongst countries, jockeying for trade advantages and/or regulated privacy supremacy.

When the GDPR is implemented, one expected outcome will be that individual nations and their Data Protection Authorities will retain a measure of autonomy to interpret and apply the GDPR’s regulation. It should be expected that although there will be a new “EU approach” to privacy-related biometrics utilization, and that there may, over time, appear to be significant and meaningful divergences in the implementation of those rules from nation to nation, within the EU. This can, and will likely complicate matters over time.

**Fig. 1:**
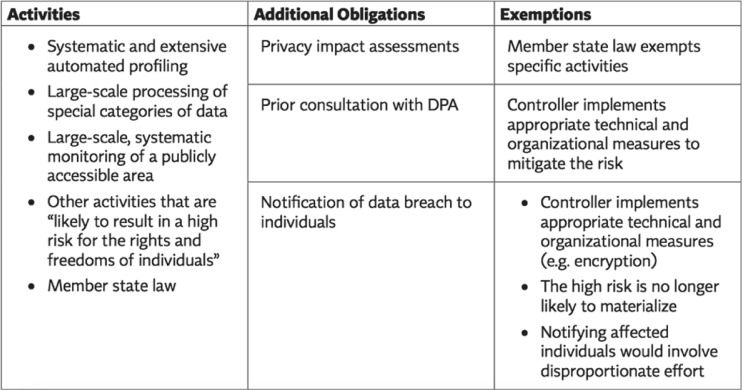
Special processing requirements and biometrics: high risk processing requirements (These requirements will be applicable to processing of biometric data.) Source: International Association of Privacy Professionals

Despite anticipated divergences, several baseline characteristics may describe EU policy regarding biometric information specifically as it is intended to operate once the GDPR comes into effect, remembering that the European approach is an omnibus approach that crosses sectors. The key tenets of the European approach toward biometric data can be broadly summarized as follows:

1. The EU requires a legal basis for the processing of personal data. Consent^84^ is one legal basis, although there are many more.^85^ These methods can get complex and arcane quickly, and are discussed in great length in other fora.^86^
2. Regarding Consent, which as discussed constitutes an important aspect for a legal basis of processing personal data in the EU, the EU Directive and the GDPR take a nuanced approach to the acquisition of Consent. Within the regulations several different types of Consent exist, each with its own standards and process.^87^
3. The processing of sensitive data,^88^ which in the GDPR for the first time includes biometrics specifically, generally requires “explicit” Consent. For a data controller to demonstrate explicit Consent, they must meet robust requirements.^89^ Fundamentally, in the GDPR, certain special categories of data are categorized as *sensitive data*; those data that reveal racial or ethnic origin, political or religious beliefs, as well as genetic, biometric, and health data are examples of sensitive data in the GDPR. Article 4 of the GDPR specifically defines Biometric data as: "'biometric data' means personal data resulting from specific technical processing relating to the physical, physiological or behavioural characteristics of a natural person, which allow or confirm the unique identification of that natural person, such as facial images or dactyloscopic data."^90^
4. But there are exceptions here too, and those GDPR exceptions cover information/data processing that is necessary for the provision of a proper medical diagnosis, the provision of health treatment more generally, or for the management of health or social care systems and services - on the basis of European Union or EU Member State law.^91^
5. Thus, the GDPR will likely allow some, or most processing of biometric data in health systems - without the giving of formal individual Consent, explicitly. But other uses of biometrics will probably not qualify for an exception, including some forms of other health-related activity (e.g., enrollment at a fitness club). Novel usage of biometrics in other contexts than those expressed in the GDPR, may require different types of Consent, or no Consent at all.
6. Regardless of the nature of Consent required for biometric information processing, the rights granted under EU law to individuals, such as rights of access, correction, and complaint, among others,^92^ will apply to data controllers processing biometric data (Fig. 1).

To summarize, any member state in Europe seeking to use an individual’s biometric data as defined in the GDPR, with few exceptions, will have Special Processing obligations under European law in regards to the collection, processing, and use of the biometric data in addition to ensuring that all other rights, such as notification of data breach, among other activities, are conducted. These are significant regulatory obligations, and provide a robust baseline of data protection and privacy for individuals. The use of digital biometric identity is widespread across Europe, with member states typically having their own deployments of digital identity systems, including those evolving from legacy (non-digital or partially digital) identity systems. Estonia, Belgium, Finland, and France, are examples of this.^93^

The European approach to biometric data differs markedly from the approach to biometric data in the Republic of India, in that India has not passed baseline data protection regulation for its digital biometric identity system, *Aadhaar*. The European approach also differs markedly from the regulatory approach of the United States, which has a sector-based^94^ approach to privacy, but nevertheless does have agreements in place with Europe, as mentioned^95^ and has privacy law touching on biometrics from a variety of sectors, as discussed next.

## US data protection and privacy regulatory framework and biometrics

The United States’ approach to biometric regulation is uneven, largely as a result of the existing US privacy regulatory structure. Unlike the EU, which operates under an omnibus data protection regulation that specifically regulates the processing of biometrics, the US operates under a complex and sometimes convoluted regulatory structure, which has been called a “sectoral framework”.^96^

In the US sectoral approach, health care, finance, education, and federal government activities, among others, are regulated separately, each with their own sets of laws and regulations at both the federal and sometimes also at the state level. This approach creates a web of federal and state level laws that --despite their volume in sheer numbers-- can nevertheless be ineffective due to substantial gaps in legal protections.^97^ Many activities lack any regulation at all in the US because the activity is not specifically included under one of the sectoral laws. Unless an activity is directly regulated within a specific sector, or under state law, it may be left out of regulatory control. Biometrics is an area that does not have its own dedicated sectoral regulation per se, but it does fall under some existing sectoral federal regulations, providing some indirect regulation, and there is also some state-level regulation of biometrics. The US is not without regulation, including biometric regulation, but the existing regulations do not do all that needs to be done in order to accomplish privacy protections on par with, for example, that of the European Union.

### Background on the US sectoral approach

Examples of US data protection law at the federal level include the health care sector, portions of which are federally regulated by the Health Insurance Portability and Accountability Act (HIPAA).^98^ In the financial sector, Gramm-Leach-Bliley Act of 1999 (GLBA),^99^ the Fair Credit Reporting Act,^100^ and the Fair Debt Collection Practices Act,^101^ provide regulation, among other laws. The education sector is partially regulated by the Family Educational Rights and Privacy Act (FERPA).^102^ Federal agency activities are subject to the Privacy Act of 1974.^103^

At the state level, states also have their own regulations that can sometimes overlap with protections provided at the federal level. In some cases, state laws can provide regulation for areas not covered under any federal regulation. For example, many states have health privacy laws that go beyond the protections afforded by HIPAA.^104^ Many states have additional financial privacy laws, for example, laws relating to identity theft.^105^ A few states have specific biometric laws, including Illinois, which has passed the Biometric Information Privacy Act.^106^ Privacy regulation at US State government agency levels is highly inconsistent, just as the state laws may also be highly inconsistent.

An additional complicating factor in the US is that federal laws may not always pre-empt state-level laws. If a federal law does not pre-empt state law, then state-level laws can provide new privacy protections in federally unregulated areas, and/or can require a higher standard of privacy for sectors already subject to some federal regulations. A good example comes from HIPAA’s interaction with state law. HIPAA is a federal regulation that provides a regulatory baseline,^107^ but States can pass laws that provide additional protections. California, for example, enacted the Confidentiality of Medical Information Act (CMIA),^108^ which provides for specific health privacy protections that go beyond what HIPAA offers. The CMIA required that California residents, who are patients, be notified of any medical data breach that involve their data, prior to the existence of any such federal standard.

One additional complicating factor to consider regarding state and federal law is that court decisions can expand or contract the interpretation of the laws, or the Constitution. Decisions can be made in civil or criminal cases. In one example of a criminal court decision regarding biometrics, in 2014, a Virginia state circuit court ruled that a criminal defendant cannot be compelled to disclose a passcode to a smartphone, noting that the passcode would be both compelled and testimonial evidence, and therefore would be protected.^109^ However, a defendant could be compelled to provide a fingerprint to open a phone with a biometric security feature, because giving police a fingerprint did not require the defendant to communicate any knowledge, and was like providing a DNA sample, which the law permits. Because this decision was made in the state of Virginia, there is some uncertainty about how it might be applied in other states.

### Key laws applicable to biometrics in the US

As stated earlier, there is not just one overarching law that applies to biometrics in the US.^110^ At the federal level, a key law that applies baseline privacy standards to the activities of the Federal government is the Privacy Act of 1974; another is the E-Government Act of 2002, both are discussed in more depth later. Another federal law, the Driver’s Privacy Protection Act,^111^ is applicable, but has extremely limited scope. Similarly, HIPAA and Gramm-Leach-Bliley can regulate biometrics when biometric data is held by a regulated institution. However, biometric data is not specifically called out, and many limitations and loopholes exist in both cases [38].

In discussing the US federal government use of biometrics, it is important to further discuss the Privacy Act of 1974. The Privacy Act is an important baseline federal privacy law. The Act covers nearly all personal records maintained by federal agencies.^112^ It applies to identity records, including biometric data held by law enforcement agencies, and it applies to military health records, veterans’ health records, Indian Health Service records, Medicare records, and health records of other federal agencies. One section of the Privacy Act also applies to state and local agencies, that is, Section 7, which requires that individuals may not be denied benefits due to non-production of a Social Security Number.

The Privacy Act passed during a time in the US when early automated computer processing created general consternation, with experts such as early computer visionary Willis Ware and others crafting the Health, Education, and Welfare (HEW) information standards^113^ that led directly to the Privacy Act and that was the inspiration for ‘Fair Information Practices,’ which later became the foundation for the EU data protection movement -- through efforts of the Organization for Economic Cooperation and Development (OECD), among others.^114^

*The Privacy Act* remains a law of substantial consequence for federal agency privacy practices, including the use of biometrics.^115^ Yet the law is rooted in many ways in the computer technology of the 1970s, and it is hard to apply to current information technology. The Privacy Act may overlap other sectoral privacy protections. For example, if a federal agency has health information about an individual, that person is entitled to the best protections in both HIPAA and the *Privacy Act.* HIPAA is better in some circumstances, but rights under the *Privacy Act of 1974* are often better than HIPAA.

*The Privacy Act* implements Fair Information Practices (FIPs), the set of privacy principles that form the basis of most global privacy law.^116^ Because it is based on FIPs principles, one of the key provisions of the *Privacy Act* is the “no disclosure without Consent” rule, also called the *Disclosure Prohibition*. Even though this might sound like it grants European-style Consent mechanisms to individuals, it is not the case. There are twelve statutory exceptions to the *Disclosure Prohibition*, including an exception for law enforcement requests. The law also includes a way for agencies to define new disclosures through a loose regulatory process, and agencies have made broad use of this authority to evade the “Consent” rule almost at will. The Act also provides for other key FIPs including accounting of disclosures, access, right to amend, and agency record-keeping requirements.

One of the most visible ways that Federal law enforcement agencies that use biometrics must comply with the *Privacy Act of 1974,* and some subsequent information privacy laws, is by publishing descriptions of their record keeping practices in the Federal Register,^117^ preparing Privacy Impact Assessments, and following other rules.^118^ Two of the best-documented examples of how the *Privacy Act* operates in a larger scale biometric system is the Federal Bureau of Investigation’s (FBI) biometric *The Integrated Automated Fingerprint Identification System* (IAFIS) database,^119^ which the Bureau says is the largest criminal database in the world with 72 million records, and its Next Generation Identification (NGI) system, which is a multi-modal biometric system including facial recognition and additional biometrics.^120^ NGI is slated to replace IAFIS. In 2017, the US Government Accountability Office (GAO) published a report highly critical of the FBI’s implementation of existing federal privacy rules in regards to its biometrics databases.^121^ One of the key criticisms of the GAO report was that there had not been the required publication of Privacy Impact Assessments prior to the development of new uses of the biometric datasets. This requirement is a key aspect of the privacy provisions of the E-Government Act of 2002.^122^

US government law enforcement agencies have requirements under *the Privacy Act* regarding how biometrics may be used to either authenticate, or to verify identity. For identity verification in particular, a hybrid approach combining machine matching, and human examination - is in use at the Federal level, in order to ensure accuracy, and to reduce the existence of high false positives. However, the hybrid approach is not always in place at the municipal level of law enforcement offices, which can lead to the improper interpretation of biometric analysis results. There has been concern regarding the use of biometrics at the municipal level in a biased and unfair manner, as well as concern regarding mission creep of Federal uses of biometrics in law enforcement areas.^123^

Another federal law affecting biometrics and identity in the US is the highly controversial REAL ID Act of 2005,^124^ which sought to strengthen driver’s license standards. REAL ID was seen by many Americans as an attempt to create a national ID system and corresponding identity database in the US due to information-sharing requirements in the law.^125^ For their part, the states saw the law as too costly to implement. As a result, the REAL ID Act has only 24 compliant states as of late 2016. In its rules implementing the REAL ID Act, the Department of Homeland Security (DHS) relaxed some provisions of the REAL ID Act to smooth over some of the objections. Regarding biometrics, the final DHS rule sets forth the minimum standards for driver’s license elements that states must include, but the rule leaves authority in the hands of the individual states as to whether to include additional elements, such as biometrics. REAL ID is still controversial today, even though it is not fully implemented.^126^ Beyond REAL ID, much of existing federal regulation relates to government use of biometric data in federal and state law enforcement activities.

In the healthcare sector, the use of biometrics requires increased attention due to the rapid adoption of technologies into the private healthcare providers settings, such as provider clinics, in the US.^127^ HIPAA, the Federal health rule, like the Privacy Act, is based on Fair Information Practices. It has two separate regulations, the Privacy Rule and the Security Rule. HIPAA would not impose any significant restrictions on use or disclosure of biometrics for many purposes (just like with other HIPAA-regulated Protected Health Information). There are no specific technical standards for biometrics, and security rules and procedures would be the same for biometrics and other forms of Protected Health Information. The health biometric playing field is open to state regulation, which would almost certainly be stronger than a HIPAA application.^128^

At the state level, there is increasing legislative activity around the use of biometrics. A Government Accountability Office report found that 41 states and the District of Columbia use biometric analysis – usually facial recognition -- to prevent fraud and abuse by driver’s license applicants.^129^ Many states have data breach legislation, and some of the states include biometrics in their definitions.^130^ However, this will simply provide notice to individuals in the case of breach of biometric or other data.

Of more interest is the direct and intentional regulation of biometric use. Illinois, Texas, and Connecticut have already passed biometric data privacy legislation.^131^ Several other states, namely California, Wyoming, and Washington State, have brought forward biometric bills, with Washington State being close to passing.

By far the most important state level law is the Illinois Biometric Information Protection Act (BIPA), which is the strongest state biometric privacy law to date.^132^ The Illinois statute requires that entities acquire consumer Consent prior to collecting biometrics; the statute applies to private entities, not the government. The original intent of the law was to prevent unconsented collection of children’s biometrics by educational institutions, but the law has had impact far beyond that. Notably, class action lawsuits based on the Illinois Biometric Information Protection Act have been brought against entities that have allegedly not gathered Consent prior to biometric use.^133^

Finally, there is also very narrowly focused legislation around the use of biometrics by children at the state level. According to the National Conference of State Legislatures, “At least 20 states have enacted legislation to protect the personal biometric information of students or minors” [41].

### Self-regulatory efforts regarding biometrics in the US

In a US-centered biometrics use context, two recent self-regulatory efforts bear examination. In 2012, President Barack Obama initiated an overarching policy program with a direct focus on the privacy of data. From within the initiative, President Obama first proposed a *Consumer Privacy Bill of Rights* (CBPR).^134^ Although the CPBR received little attention from Congress, the Consumer Privacy Bill of Rights relied on Fair Information Practices, as well as the concept of contextual privacy, as theorized by Helen Nissenbaum [42].

Three Multi-Stakeholder Processes (MSP) convened by the US Department of Commerce through the National Telecommunications and Information Administration (NTIA), and beginning in 2012, were part of the Obama privacy initiative. The general goal of the MSP was to forge a different way to develop privacy self-regulation. The process envisioned:

*Open, transparent forums in which stakeholders who share an interest in specific markets or business contexts will work toward consensus on appropriate, legally enforceable codes of conduct. Private sector participation will be voluntary and companies ultimately will choose whether to adopt a given code of conduct. The participation of a broad group of stakeholders, including consumer groups and privacy advocates, will help to ensure that codes of conduct lead to privacy solutions that consumers can easily use and understand. A single code of conduct for a given market or business context will provide consumers with more consistent privacy protections than is common today….* [43]

The focus of the first effort was mobile application transparency, specifically, short form privacy notices for mobile devices such as smart phones. In public meetings, consumer and privacy groups worked together with industry and trade associations to develop a consensus “code of conduct.” The product of the process after a year and a half of work was the “Short Form Notice Code of Conduct to Promote Transparency in Mobile App Practices.”^135^ The notice established several privacy benchmarks, including being one of the the first US model notices related to the issuance of notice to users regarding biometric use, albeit a voluntary, non-binding model notice.

In 2014, the Department of Commerce commenced a second “multi-stakeholder process” on the topic of commercial facial recognition.^136^ Even though discussions in the area began in February 2014, over some time, the advocacy communities and industry stakeholder representatives failed to find common ground, despite overall engagement in the discussions. The first point of contention was that prior to the discussions, the parameter of the discussions were not to include government use of biometrics, a requirement from the NTIA that the advocacy groups generally did not agree with, and were given no opportunity to dispute. During the discussions, a second key point of contention was the role of consumer Consent to the collection of biometric information in commercial activities. Industry representatives did not concede that consumers had any relevant role in the issuance of Consent related to Biometric information collection or use. Therefore, in June 2015, after more than a year of meetings, the privacy, civil liberties, and advocacy groups staged a well-publicized walkout, formally abandoning the NTIA facial recognition stakeholder process.^137^ Industry representatives, and the Obama Administration - continued on with the process, without the consumer groups’ input or involvement.

The public interest groups wrote, in part:

*At this point, we do not believe that the NTIA process is likely to yield a set of privacy rules that offers adequate protections for the use of facial recognition technology. We are convinced that in many contexts, facial recognition of consumers should only occur when an individual has affirmatively decided to allow it to occur. In recent NTIA meetings however, industry stakeholders were unable to agree on any concrete scenario where companies should employ facial recognition only with a consumer’s permission*.^138^

For its part, the Obama Administration commented that:

*Multi-stakeholder processes work best when a broad range of stakeholders with differing viewpoints fully participate. Most importantly, stay in the room.* 139

The final outcome of the NTIA facial recognition proceeding remains murky, however a commercial sector code of conduct now exists, antithetical to the perspective offered by privacy advocates. Regardless, and for the purposes of this article, there are selected points that are worth noting from the NTIA exercise. They are:

- The process of US consumer groups working with the US biometrics industry did not go well in this iteration, and Consent was the crux of the issue that caused the talks to fail. Neither side was willing to compromise their positions regarding Consent.
- The talks were focused on one aspect of biometric use; facial recognition in the commercial context. It would have been much more productive to address a specific use case versus a specific technology.
- Attempting to have a conversation about biometric use that does not include government use cases is unrealistic.
- Self-regulation discussions need to be both structured and thoughtfully managed in order to reduce breakdowns in discussions. Part of this will include making factual, evidence-based decisions, versus decisions based on mere rhetoric.

This conversation in the US took place while the EU was in the midst of negotiating the GDPR legislation. It is unknown if the failure of the NTIA talks led to a more stringent EU Consent requirement for biometrics. The NTIA facial recognition effort was a high-profile policy failure, nonetheless. With a better structure for discussion, more willingness on the part of all participants to find a middle ground, and a more specific use case to discuss, the outcome may have been different.

Although the US is a high-income country, its approach to biometric regulation is not as protective as the European Union. The US approach does not offer enough regulatory protections to guide the increasing uses of biometrics. While the Privacy Act of 1974 should theoretically provide protection in the case of Federal government uses of biometric technology, the US Government Accountability Office (GAO) report on the implementation of Federal privacy was not encouraging regarding compliance and transparency in uses by law enforcement.^140^

In the majority of countries, authority for identity and/or privacy falls to a designated office of either an identity authority, or a data protection authority. The US does not have a specific identity authority, nor does it have a formal data protection office. While the US Federal Trade Commission (FTC)^141^ is tasked with enforcement of some consumer protection laws in regards to unfair and deceptive business practices, by no means is the FTC a full-fledged data protection authority. And while the US does have a Department of Transportation (DOT),^142^ the DOT is similarly not a full-fledged identity authority that has legislation mandating it manage the integrity of the identity of its citizens as its primary focus.

The US has not supported the idea of a national digital identity scheme thus far, and the negative reaction to REAL ID is an indicator that further development will require a more privacy-protective legislative approach to the issue. However, it is unlikely that over the long term the US will be able to be one of the few remaining countries in the world without some form of national digital biometric identification, which means there is much work to be done regarding biometric policy and privacy protections in the US at the federal and state level.

## Discussion: biometrics policy

Of the three jurisdictions discussed in this paper, each has a completely different framework for the data protection and privacy of biometrics. In the Republic of India, the *Aadhaar Act* and other legislation does not provide comprehensive data protections and privacy for the *Aadhaar* program and its use of biometric data.

The EU, as discussed, has an omnibus data protection and privacy policy that is comprehensive and also includes specific language regarding biometrics processing, including automatic processing. As such, the EU has protective data protection and privacy regulations already in place for any member country that builds or employs biometrics, or more broadly, a digital biometric identity system.

The U.S. has a patchwork of focused, sector-based regulation that applies unevenly in regards to data protection and privacy for biometrics, including a lack of broadly applicable data protection and privacy legislation on the use specifically of digital biometric identity systems. While the REAL ID Act does include some aspects of identity systems, it leaves biometric use up to the states, and therefore does not act as a unifying regulatory framework for biometrics or for all digital identity systems. Additionally, the REAL ID Act is not a data protection regulation, nor was it meant to function as such. In the US, some data protection for biometrics comes from the Privacy Act of 1974, which has numerous exceptions, some comes from sectoral law, such as HIPAA, and some comes from state law, which is very limited in scope at this time. In order to further analyze India’s approach to biometric policy and privacy, it is useful to investigate a central issue area of biometric policy, which is that of Consent. 

### Consent and biometrics

Consent is a core issue in regards to biometrics and identity, and amidst the myriad potential issues, Consent is readily among the most contested of them. If there is no fundamental Consent for individuals regarding biometrics and identity, then autonomy and human freedoms can be at risk, depending on existing protections, and how well those protections are enforced. As with the differing standards for privacy, there also is no single standard, global definition in use for Consent regarding use of biometrics. Additionally, “Consent” is simply one small practitional aspect within a much larger framework, needed to assure data protections generally, as well as specifically according to standards such as OECD’s Fair Information Practices.^143^ But it is a particularly important aspect, as it affects voluntariness and issues of autonomy. (As discussed elsewhere in this paper, Fair Information Practices provide the baseline for most global privacy law, and although the principles do not cover all privacy rights, it is a globally accepted baseline.^144^).

In India, the *Aadhaar Act* and other existing regulations do not provide robust Consent provisions in regards to the collection of biometrics; it should be noted that the Act stands in opposition to the India Supreme Court interim decision regarding voluntariness,^145^ a decision that *Aadhaar Act* contravenes. The provision in Indian law that Consent can be accomplished “through any electronic means” leaves substantial loopholes through which, the broad principles underlying Consent, and all associated processes can be trivialized. This is a foundational problem in India regarding *Aadhaar* and Consent.

Considering health use cases in India specifically, healthcare information is deemed to be sensitive data under India sectoral law.^146^ India’s healthcare biometric landscape has a high total numbers of users; as discussed, more than one billion, and now *Aadhaar* is tied to increasing numbers of medical programs. Because *Aadhaar* enrollment is now mandatory to receive most government benefits, and because well over 80% of the population is in the *Aadhaar* system, national health policy has incorporated, and expects *Aadhaar* information to be input into the medical system, which includes a new e-Health system tied to mobile phones.^147^

When enrollment and possession of the *Aadhaar* is made mandatory, in government benefits and other settings, and when the *Aadhaar* activity is linked to many aspects of individuals’ lives over a lengthy span, all located in one centralized database, Consent becomes a highly significant issue. Ideally, well-thought through policies need to be in place to provide meaningful checks and balances for individuals. In India, the early emphasis has been on reducing inefficiencies, not on protecting privacy or autonomy. The loss of autonomy regarding Consent has been deeply felt, and now needs to be addressed.

One example of the difficulty of making *Aadhaar* mandatory for health services is in the newly-mandatory use of *Aadhaar* for women and others in India who are being rescued from prostitution, who cannot receive rehabilitative services until they have enrolled in *Aadhaar*. One prominent legal scholar said the anonymity of these women was the first casualty.^148^ Due to the social structure and other factors in India, women and others may have been born into prostitution, or may have been the victims of human trafficking. Those who want to be rescued from that life already have many hurdles to overcome, not the least of which is social stigma and shame^149^; the requirement of loss of anonymity in seeking health services adds to the obstacles facing these individuals, and is not acceptable on a human level.

The Council of Europe’s *Convention on Action against Trafficking in Human Beings* specifically discusses the need to protect the private life of and identity of victims, including victims who are children.^150^ Rijken and Koster (2008) argue that victims of trafficking must be provided with specialized medical care as well as legal aid, and need to be given assistance regarding the “juridical consequences of filing a complaint and testifying against perpetrators.” They also discuss in detail the extent to which identity documentation plays a role in acquiring testimony against the perpetrators for state purposes. The authors advocate a “victim centered approach,” where the goals of granting robust assistance to victims first and foremost take precedence over the goals of government in identifying victims [47].

But these vulnerable individuals are not the only casualties of coerced Consent for *Aadhaar* in India. For example, in 2016 the state of Maharashtra mandated that the *AEBAS* (*Aadhaar* Enabled Biometric Attendance System, which is connected to all central government offices) be used in all government-run hospitals in the State. This requirement applied to health workers. Numerous articles about problems and negative reactions among health workers across India have been published. In one hospital, 22 doctors refused to use the biometric attendance system, and by way of protest, were absent from duty, [48] alleging that the system was discriminatory. One issue was that the *AEBAS* system allows for real-time attendance data to be stored in the *Aadhaar* central database, which employees and officials can view [49].

The privacy challenges in such a detailed, centralized, transactional database open to external government and employer access are significant. Note the fundamental differences between allowing for biometric authentication in a small silo, not tied to an extensive identity database of life patterns, and that of binding biometric authentication to *Aadhaar* – while linking the work check-in for instance, to the rest of an individual’s life activities such as banking, health, marriage, and more. Even though biometrics are involved in both instances, the privacy implications are different. In the India example, there is simply no fundamental privacy redress for affected individuals, and the issue of a lifelong, government-controlled, central tracking database of life, financial, health, and work activities is something that fuels the darkest of Orwellian fears.^151^ If specific regulations constraining uses of the biometric system and centralized database are absent, new -- and mandatory -- uses will simply grow, based on what has already been seen in the *Aadhaar* system.

The mandatory *Aadhaar* checkins by physicians are an example of ‘Coerced Consent,’ which arises in situations where an individual believes, is led to believe, or is allowed to believe - that in order to receive a perceived benefit, that he or she must Consent. In the EU, coerced Consent is a policy issue addressed by law.^152^ In US Consent policy, the subject of ‘Coerced Consent’ is discussed in selected areas handling high sensitivity matters, which are often related to the use of genetic information in labor situations, or medical research. For example, the following FDA statement relates to patient Consent, and the issue of coercion:

*Consent documents should not contain unproven claims of effectiveness or certainty of benefit, either explicit or implicit, that may unduly influence potential subjects. Overly optimistic representations are misleading and violate FDA regulations concerning the promotion of investigational drugs [21 CFR 312.7] or investigational devices [21 CFR 812.7(d)] as well as the requirement to minimize the possibility of coercion or undue influence [21 CFR 50.20].* 153

Note that the FDA’s conception of consent describes the high quality of the information needed for those making the consent decision. This is foundational to consent that is well-educated by facts, thus creating the ability for an individual to make an informed consent decision.

“Coerced Consent” is going to need to be on the policy watch-list globally. Reducing inefficiencies, including in health care settings, should not come at the expense of conditioning a person’s employment on having an enrolled biometric, or for that matter, provisioning treatment on the production of identification. Other options can, and should be made available, so as to avoid such outcomes, both in technical and policy solutions presented. While the gaining of Consent in biometric use cases is critical, such Consent given does not then translate to a blanket protection of privacy, however, such Consent gained has a proper place in asserting biometric policy.^154^

Regarding biometrics-specific consent policies, in the United States, specific biometrics Consent policy exists just in State law. In the European Union, (and those nations with current EU adequacy status),^155^ the GDPR and to a lesser degree, the conventions of the Council of Europe (COE)^156^ have ensured that “Consent” will be a meaningful part of biometrics deployment specifically, after the 2018 implementation of the GDPR. In Europe, obtaining Consent in general is the basis of most privacy and human rights-focused laws, decisions, and discussion. Obtaining “Consent” has been a critical thread in the fabric of national European data protection laws, since the 1970s, with the role of Consent continually evolving toward more stringent standards. Consent was eventually recognized in the European Charter of Fundamental Rights, Article 8(2), which states that personal data of an individual can be processed “on the basis of the Consent of the person concerned, or some other legitimate basis laid down by law.”^157^ Given this strong legislative background, it is not surprising that biometrics gathered from data subjects would eventual warrant specific Consent requirements.

The new GDPR requirements for Consent include the requirement that the consent be informed; speaking broadly, there can also be applications regarding Consent for the use and processing of sensitive data. Biometric data as defined in the GDPR is considered sensitive data, and therefore, will require Consent as part of the sensitive data category.^158^ Additional privacy provisions would still apply around the processing aspect of the biometric data.^159^ The older EU 95/46 standards were interpreted by Article 29 Working Party^160^ at length, and included an analysis of Consent in the context of e-cards, which is worth reading in the context of biometrics even though this law will be replaced by the GDPR, because it lays out the foundational EU ideas about Consent in data processing and in sensitive data categories.^161^

Terms of the GDPR state that all biometric use conditions will require special processing under the sensitive data category.^162^ There are however, exceptions, including in certain health care areas, and based on the definition of Consent in the GDPR. The primary impact of the EU decision to include biometrics data as a sensitive data category in the GDPR is bound to have profound policy impacts in the biometrics world. The impact will be most keenly experienced by entities based in, or doing business with, Europeans. The GDPR biometric policy will also impact any company self-certifying under the EU-US Privacy Shield/Swiss-US Privacy Shield,^163^ because these companies will have to follow GDPR provisions regarding biometrics.

The study of biometric use and interactions within Europe’s Consent model, particularly in the healthcare sector, can be deemed to be important. The use of biometric systems for the identification of patients has already begun in Europe. Healthcare providers within individual EU member countries, for example, Ireland, are introducing the use of biometric into health provider settings. A typical scenario is that patients will enroll in the biometric system, and provide personal biometric information, for the stated purpose of identity verification, in relation to their record and for anti-fraud purposes. Healthcare providers in EU member countries will have to comply with GDPR requirements in 2018, including those who provide allied services in healthcare settings, which will require attention to processing controls.^164^

In Europe, if a health care provider requires a patient to enroll in single-provider biometric silo (which they can do), patients in EU settings should, on the basis of both the existing Data Privacy Directive and the GDPR, receive other supporting privacy rights, such as access, transparency, and correction. And the processing of the biometric data will still have to comply with all applicable EU standards. Although protections will exist due to EU omnibus privacy regulations, prior to any further dispersions of healthcare biometric installations, EU member states would greatly benefit from encouraging respective EU healthcare sectors to devise specific ‘best practices’, and ‘ethical data use’ guidelines.

The US does not have any consolidated regulatory framework across sectors focused only on biometric Consent policies. As discussed earlier, some laws touch on biometrics held by sectoral entities, like the federal government. But sectoral laws, like the Privacy Act of 1974, do not mention biometrics specifically. The only specific law regarding explicit Consent for biometrics is currently at the state level, for example, the Illinois state law BIPA requiring Consent specifically for biometrics collection. BIPA, however, does not have a complex Consent policy. To find mature Consent policy examples in the US, one has to study policy assertions apart from biometrics. The US Food and Drug Administration (FDA) has a detailed description of Consent, for example, which specifies all that must be done to ensure that the Consent is meaningful, voluntary, and not coerced.^165^ Generally, any federally-funded entity falling under the Common Rule,^166^ is going to display a Consent policy, at the most sophisticated of levels. However, such presentation of a Consent policy could not be interpreted, either directly, or indirectly, as a Consent policy that would fully cover, or apply to the use of digital biometric identity in any simple or straightforward way.

When biometrics are used in non-research healthcare settings for authentication or identification, generally the Consent documents for human subjects research rules do not apply. This is because research Consent documents are generally not required for non-research healthcare provider activities, and research Consent documents are focused on the actual health research, not the identity documentation of the patient or research subject. It is a gap in the regulatory structure.

Consent has become a point of contention in US health care settings that require biometric enrollments for patients. In Florida, a 2016 bill was put forward that would have required that hospitals “biometrically confirm the identity of Medicaid patients.”^167^ The proposal would have allowed hospitals to access the state driver’s license database to verify patient driver’s license identification. The Florida Hospital Association opposed the provision, and raised substantive legal and privacy concerns [51]. Public hospitals in the US are prevented from mandatory biometric requests due to laws preventing provisioning of treatment based on identification. Biometrics installations at private US healthcare providers such as private hospitals may not be subject to the same requirements, however.^168^ Some healthcare providers in the US have strongly urged patients to provide biometric-based authentication or verification, with apparently little attempt made to ensure patient knowledge of voluntariness of enrollment. [52] There is currently a policy void regarding this issue, which is, by itself, significant.

Intriguingly, in the US, biometric identification of patients has broadly been put forward as a “solution” to challenges, such as identity theft associated with the provision of medical services [53].^169^ Identity theft challenges apply to Europe as well. However, discussion of biometric template takeover, spoofing (or falsifying) of biometric identity, full biometric identity takeover, data breach risks, and other significant complications to the patient biometric systems, are almost never included in discussions around implementations [55].^170^ Weak security and policy understanding of biometric technology can create weak oversight situations where imposters have an opening to harden a spoofed or acquired false biometric identity.^171^ It is rare to find straightforward risk/benefit discussions related to patients’ biometric identifiers - including in relevant Notice of Privacy Practices (NPPs). It is also rare to find media articles mentioning problems with biometrics security in healthcare settings in the US. Later in this article, untraceable biometrics are discussed as an important area for future work that could help attenuate some present and future challenges in this area.

In thinking about India’s Consent policies in the context of those in the EU and the US, particularly in a health care use context, each jurisdiction does have some legislative language around Consent and the Sensitivity of Health Data. However, how the legislative language is contextualized in terms of definitions of Consent and procedures is what separates the jurisdictions in available privacy protections. Ultimately, the significant inter-links of the *Aadhaar* and the tracking of enrollees’ activities in a centralized database with extensive government capacity for access to that database are unparalleled in any other legal jurisdiction discussed in this paper. Mandatory biometrics use propositions in India need to be addressed directly and with some urgency, and especially so in the health services context.

### Biometric legislation

In the case of digital identity systems, formal data protection and privacy legislation is a must; voluntary guidance or voluntary principles are not an acceptable substitute.^172^ The same can be said of digital biometrics identity systems. Among current regulations, the EU GDPR provides the highest level of current protections. Other legal jurisdictions generally have either weaker protections, or no protections at all.^173^ India has not passed data protection regulation, although it has drafted such legislation. As discussed, the US has some federal and state legislation that touches on aspects of either identity or biometrics, and sometimes both, as in the REAL ID Act; however, the US does not have specific, focused federal legislation around the broad use of biometric data.

In non-EU jurisdictions, much progress is possible if serious attempts at legislation aimed at improving data protections and privacy specifically for biometrics use, including digital biometric identity data, are undertaken. There is no doubt that economic and cultural differences impact deployment of digital identity systems and biometrics as well as policies around those systems. The US, for example, will have to take a different approach to legislation than India based on multiple factors such as the structure of existing federal legislation and the state of development of biometrics in each country. However, that is not enough of an excuse for the US and India to avoid working on the challenging issue of passing new legislation. In India in particular, because the *Aadhaar* is already pervasive and used in a central database, data protection and privacy legislation specific to *Aadhaar* is important, and urgent, for India to put in place.

Generally, low income, middle income, and high-income countries have different levels of development and may not be able to physically support the same kinds of technologies, systems, or policies. Some countries may not have the same cultural conceptions of individual privacy rights. Nevertheless, despite the many types of legislation that might be appropriate for any given economic jurisdiction or region, several core legislative concepts stand out. These concepts may be used across cultural and economic boundaries.

#### Do no harm

Digital biometric identity systems have power, and once granted, that power can be used for good or otherwise. Adding biometrics to an identity scheme (digital or paper-based) simply increases the power of the identity scheme by increasing belief in the accuracy of the system to be able to uniquely identify or authenticate a person. As such, the *Do No Harm* mandate is of primary importance in all identity systems, particularly those using biometrics. The joint *ID4D Principles on Identity* have been discussed in this paper. These principles are important because they are aimed at developing countries; fortunately, these principles do indeed include principles relating to privacy and non-discrimination. However, they do *not* include a *Do No Harm* principle. It is the most important missing element of the principles, and the addition of *Do No Harm* to these principles is of great importance and would improve the principles considerably.

What constitutes harm? Different political, economic, and cultural contexts exist for digital biometric identity systems, so it can be expected that different types of harm will arise, each unique to the system that it is situated in. In practice, *Do no Harm* means that biometrics and digital identity should not be used by the issuing authority, typically a government, to serve purposes that could harm the individuals holding the identification. Nor should it be used by adjacent parties to the system to create harm.

Examples of harm include identifying highly sensitive divisions amongst populations (such as ethnicity, religion, or place of origin). Just by attaching that data to a unique biometric is a substantive harm in and of itself. To use an identity system to discriminate against, harass, deny services improperly, or otherwise cause harm based on distinctions such as age, gender, or socioeconomic status as revealed by a place of residence constitutes harm. In India, it is a great harm existing today to provision the delivery of rehabilitative services to women and others attempting to escape prostitution on having been enrolled in the *Aadhaar* program. As discussed in the Consent section of this paper, the *requirement* of loss of anonymity in seeking rehabilitative or health services adds to the obstacles facing these individuals and is not acceptable on a human level.^174^

Another type of harm can arise from the politics of identity. Some identity systems have been tied to the politics of a government or an ethnic faction of a government. It is very difficult to de-link identity systems from the government that issues the ID, but every effort should be made to de-link e-ID systems from the politics of the government or faction in power.^175^ A disturbing political use of identity cards is found in the haunting case of Rwanda. It is widely acknowledged that Rwanda’s ID card, which included ethnicity on the face of the card, was used to facilitate mass genocide against the Tutsis in 1994 [3, 58]. This is the ultimate harm, and all efforts should be taken to avoid it in the future. Identity systems, no matter what form they come in, paper or digital, must work for the public good and must do no harm. And identity systems, due to their inherent power, can cause harm when placed into hostile hands and used improperly. Great care must be taken to prevent this misuse. *Do No Harm* requires rigorous evaluation, foresight, and continual oversight.

#### Policy before technology

More than any other factor, the underlying cause of India’s current problems with *Aadhaar* are a result of the lack of appropriate regulation of the *Aadhaar* ID system before its widespread deployment into the Indian population. Legislating in reverse is extremely difficult. When the technology for the *Aadhaar* system -- including the collection of biometrics -- was discussed as a potential program, legislation regulating the targeted and limited use of the *Aadhaar* identity and data should have been put forward as a mandatory step prior to any widespread technical deployment or biometric enrollment of residents. As discussed in this article, although several iterations of acceptable privacy legislation have been drafted in India, including in 2010 as the technology was being initially deployed, none of the legislation has passed. The lack of protective policy from 2010 onward has allowed the *Aadhaar* ID to go from voluntary to now mandatory in many situations without appropriate data privacy protections. As of today, the *Aadhaar* ID system is subject to considerable mission creep, and there are concerns about how it might be used in the future. It is very unclear if India will pass data protection legislation for the *Aadhaar* system.

When advanced digital biometric ID systems are discussed, Estonia is frequently cited as an examplar of a modern digital identity system in addition to *Aadhaar*.^176^ However, the two systems are different. Estonia, as a member of the European Union, already had a robust policy system in place before it put its e-ID, or digital identity, technology system in place. Because of the underlying EU data protection and privacy rules, Estonia is obliged to comply with all EU law, including EU data privacy directives. Estonia’s e-ID will fall under the GDPR biometric processing protections and mandates discussed in this paper, and it will be subject to other sensitive information categories. Estonia’s e-ID system has an omnibus set of legislative rules to follow, including privacy rules, data security rules, redress rules, and many more. Estonia had *policy before technology*, and that has made it a fairer system, not subject to the same abuses as India’s *Aadhaar* system, which put technology before policy.

The US is not immune to challenges arising from the “policy before technology” issue. In Federal agencies, the E-Government Act of 2002 requires “policy before technology” evaluations – for example, agencies must publish Privacy Impact Assessments (PIA) for public review prior to developing, procuring, or creating new uses of technologies [59]. This is beneficial, as future uses of biometric technology at the federal level that are proposed should conceivably be made public prior to their installation and use. However, this is limited in that Privacy Impact Assessments (PIA) are published regarding government uses of technologies; also, the publication of a PIA does not guarantee that a bad program will not move forward. The US, as discussed, has widely deployed biometrics in non-federal sectors such as healthcare. Almost all of these deployments have occurred without specific biometric legislation preceding the deployment of the technology. As discussed in this paper, there is no federal law that protects biometric data specifically collected for example, by schools, hospitals, commercial entities, or other non-federal entities. And when a US federal agency delays its publication of a Privacy Impact Assessment, it makes it nearly impossible for individuals to assess what the federal government is planning.

#### The role of ethical data use guidelines for biometrics

In addition to formal legislation, it would be beneficial for all stakeholders --industry, privacy and civil liberties NGOs, identity experts, academics, and interested citizens and individuals --- to convene as stakeholders in order to craft “ethical data use guidelines” under the support of a well orchestrated multi-stakeholder process. These guidelines could, for example, cover very narrow use cases where regulatory rules presently do not offer specific guidance related to best practices, conceiving and establishing procedures, and administrative controls. For example, a specific set of “ethical data use guidelines” regarding the collection of patient biometric data by health care providers could be made to emerge useful practical guidance - in addition to the formal protections of the GDPR.

An important policy document to consider comes from the European Data Protection Supervisor (EDPS), which, in 2015, published a watershed opinion regarding data ethics and privacy.^177^ The opinion set forth four overarching principles:

1. *Future-oriented regulation of data processing and respect for the rights to privacy and to data protection.*
2. *Accountable controllers who determine personal information processing.*
3. *Privacy conscious engineering and design of data processing products and services.*
4. *Empowered individuals.* 178

The opinion specifically triggered the launch of a new EU Data Protection Ethics Board - with the goal of defining “new digital ethics” and stimulating “open and informed discussion in and outside of the EU, involving civil society, designers, companies, academics, public authorities, and regulators.” The opinion sets out in clear terms the next steps that could and should be taken regarding biometrics policy. In many contexts -- more applicable to jurisdictions outside the EU than inside the EU -- there exists interest to support the presence of such discussions. Structural and financial support for such activities will need to be put into place, or support will need to be provided by the EU Central Authority, or by other countries.

However, for long-term success to occur, rules and procedures need to be in place that provide ‘checks and balances’ to ensure input and process control, enforcement, and representation of interests.^179^ The National Consumer Council in the UK published an important 15-point checklist for self-regulatory schemes in 2000 that remains worthy of attention [62]. The checklist offers requirements for a “credible” self-regulatory scheme. These same principles, although initially written as applicable to self-regulatory schemes, can also apply to multi-stakeholder processes with the stated purpose of crafting ethical data use guidelines.

Despite the potential for failure, [56] it is nevertheless important for industry and consumer-focused stakeholders to convene, allowing each stakeholder to put forward an independent contribution, in order to look at multiple, narrow use-case scenarios regarding biometrics use and data ethics. In many respects, ethical data use guidelines for very narrow use cases have more possibility of success, particularly when approached from narrow use cases. One example of a narrow use case is ethical data use guidelines for biometric health identity data used in formal health care settings, such as a hospital or doctor’s office. In all jurisdictions, one important use case could be on ethical data practices around particularly sensitive ethnic data.

It would, over the long term, be helpful to have open, joint stakeholder discussions amongst countries with large-scale biometrics installations so as to share solutions, findings from relevant encounters, amassed expertise, discuss concerns and challenges, and engage in forward-thinking policy construction^180^ relating to ethics, data protection, and privacy. The idea of crafting ethical data use guidelines in the area of privacy would need to be inclusive of standards, which could differ markedly depending on geography, Fair Information Practice standards (FIPs),^181^ key provisions in the GDPR, the ID4D Principles on Identification, among others could potentially be discussed. Other types of standards that could be drawn from could include very precise standards from the ISO, which would include, for example, the standard on cross jurisdictional and societal aspects of biometrics, JTC 1/SC 37/WG 6, or identity management and privacy technologies, JTC 1/SC 27/WG 5, by way of example.

#### Privacy by design

Digital identity systems and systems that use biometrics need to be designed in such a way that they cannot fail, even when political regimes and the will of legislators do [63]. This core concept, derived from the Privacy by Design school of thought,^182^ is particularly important in the case of biometrically-enhanced digital ID systems. If an individual can be uniquely identified by a strong biometric like an iris scan, there is a great burden on the designers of that system to ensure failsafes for the individuals who hold that identity. This kind of design is becoming more technically possible, but there is not yet a deployment that would sufficiently protect identity holders from abuse of the identity by those in power. All jurisdictions would benefit from an approach that considers privacy by design in biometric identity systems. However, it is important to note that while all jurisdictions would benefit from an approach that considers privacy by design in biometric identity systems, it should not be seen as a substitute for legislation or other protections.

The technique of biometric encryption and “untraceability” provides a starting point for the kind of privacy by design work that might ensure that an digital ID or other biometric use could not be misused by a government in power, or a company. Ann Cavoukian, former Privacy Commissioner of Ontario, Canada, when in office had the prescience to craft and adopt a policy for biometric technology use in the late 1990s [66]. The protections are remarkable for their time and include use of untraceable biometrics supported by policy. This came about when the City of Toronto wanted to install biometrics use in order to reduce fraud in public services. Commissioner Cavoukian crafted a policy proposal for the government, and urged formal legislation to enshrine those practices.

The IPC proposal stated the following:

*The biometric (in the case of the City of Toronto, it was a finger scan) should be encrypted;*

*The use of the encrypted finger scan should be restricted to authentication of eligibility, thereby ensuring that it is not used as an instrument of social control or surveillance;*

*The identifiable fingerprint cannot be reconstructed from an encrypted finger scan stored in the database, ensuring that a latent fingerprint (that is, one picked up from a crime scene) cannot be matched to an encrypted finger scan stored in a database;*

*The encrypted finger scan itself cannot be used to serve as a unique identifier;*

*The encrypted finger scan alone cannot be used to identify an individual (that is, in the same manner as a fingerprint can be used);*

*Strict controls on who may access the biometric data and for what purposes should be established;*

*The production of a warrant or court order should be required prior to granting access to external agencies such as the police or government organisations;*

*Any benefits data (personal information such as history of payments made) are to be stored separately from personal identifiers such as name or date of birth.*

The Social Assistance Reform Act of Ontario, Canada was passed in 1997.^183^ The legislation required the following:

- That biometric information collected under the Act must be encrypted;
- The encrypted biometric cannot be used as a unique identifier, capable of facilitating linkages to other biometric information or other databases;
- The original biometric must be destroyed after the encryption process;
- The encrypted biometric information only can be stored or transmitted in encrypted form, then destroyed in a prescribed manner;
- And, no program information is to be retained with the encrypted biometric information.

The final legislation also included a specific provision that the full gamut of administrators of the biometric system could implement

*a system that can reconstruct or retain the original biometric sample from encrypted biometric information, or that can compare it to a copy or reproduction of biometric information not obtained directly from the individual.*

While the final regulation was not as complete as the initial IPC recommendations, it stands as a groundbreaking and forward-looking piece of biometric regulation. The regulation is important for its technical protections combined with the policy protections of not allowing for biometric reconstruction or transactional tampering. Additionally, the legislation kept the data in a localized “silo,” requiring that the data not be networked into other databases or a larger system, thus keeping linkages from occurring. For example, the social assistance data would not be readily accessible by potential employers. The City of Toronto achieved its goal of reducing fraud, and the IPC achieved its goal of protecting consumer privacy.

Today many potential opportunities exist to use technical biometric protections in a way that enhances consumer privacy, dignity, and autonomy. However, the best practices, knowledge, and discussion must be public, ongoing, and robust in order for this to occur.

Many additional principles for legislation exist. This has been by no means a complete list. OECD Fair Information Practices, Europe’s GDPR, the *ID4D Principles on Development*, India’s *Group of Experts’* report, and the Do No Harm principle – all of these stand as important sources for legislative guidance in the area of digital biometric identity.

## Conclusion: what are the stakes for a failure to act?

In considering India’s *Aadhaar* program and its lack of adequate protections of privacy and autonomy, what stands out the most is the continuum of choices that have to be made to protect privacy rights, freedom of choice, and how the *timing* of making the right choices appears to matter a great deal. India’s *Aadhaar* deployment put technical deployment before policy development, and continued to do so. These actions by the government of India have led to a marked lack of protective regulatory controls for the *Aadhaar* program, which has in turn resulted in profound mission creep and a loss of autonomy. India is a case in point that by the time a deliberative legislature can move a thoughtful bill to passage, a fledgling biometric program may have attained pervasiveness, and thus be very difficult to regulate or remove in backwards motion.

Now, with 97% of adults enrolled in the *Aadhaar* biometric scheme in India, India’s policy around its government-issued national biometric identity card may have garnered benefits, but it is also riddled with highly problematic human rights and other challenges. The mission creep and data linkages around the *Aadhaar* identity number are a high priority to address. Begun as a voluntary identity card, now Indian residents cannot even buy a train ticket without an *Aadhaar* number, nor can they marry, purchase or own property, or teach; soon banking records and medical records will be tied to the central identifying *Aadhaar* scheme.

In the name of efficiencies or modernization, is it appropriate or desirable to link life activities to a central government database, one without vigorous privacy protections, and without significant constraints on government access to that data? It has now been since 2010 that *Aadhaar* has been in place, and since 2016 since the Indian government has begun greatly expanding *Aadhaar* linkages. The time is growing short for India to address the problems with *Aadhaar;* It is not yet clear if a future generation of India’s policymakers will push *Aadhaar* policy back into a more constrained set of boundaries, ones which would allow for reduced linkages and much greater *voluntariness*, transactional privacy, and freedom of choice while still retaining benefits. If uses are left to expand uncontrollably, the *Aadhaar* system could turn into a golden key that could have far too much unchecked control over citizens.

In contrast to India, a close review of Europe’s approach in the GDPR reveals it to be a bold effort to protect digital privacy in digital ID systems. While the introduction of biometrics to sensitive data categorization surprised many in other countries, it was the right choice made at the right time to protect human rights during a time when biometric deployment will increase. Much rests on Europe’s “privacy firewall” to extend a positive influence on other jurisdictions.

For its part, the US system does not have effective, specific legislative protections at the federal level regarding biometrics. It has limited areas of protections, and the trickle of state law activity could, if increased, serve to bolster protections in some limited areas of biometrics use, but that will not be enough by itself. It is unclear what pathway the US will eventually take regarding biometrics and privacy. But given the increasing deployment of patient biometric authentication in health care settings, and the high potential of a national digital biometric identity system in the future, the US will need to pay close attention and take focused action in order to address the forthcoming and significant security and privacy challenges.

Going forward, the hope is that smart regulators will heed the warning bells and enact reasonable, privacy-protective legislation now. If there is one key lesson to be learned, it is that policy development needs to focus on the concept of *Do No Harm*, and policy should come before technology deployment whenever possible. When it has not been possible prior to the launch of technology, then policy development needs to be a top-line priority thereafter.

Biometrics have the ability to create trusted identities, and where that exists in digital, transactional ecosystems, a high degree of risk to fundamental civil liberties and privacy also exists. It is simply not possible to have a digital ID with biometrics that does not create fundamental risks of surveillance, risks of social and or political control using the system, and the risk of pervasive privacy violations. No matter what the level of economic or legislative development exists for a region, *Do no harm* must be the bedrock guiding principle of all digital biometric identity systems.

## Footnotes

1 The World Bank Group maintains a list of all jurisdictions and the development levels of identity documents and systems. The data is available for download. See: [1]

2 Boersma et al. [4] See pages 170–185. Available at SSRN: https://ssrn.com/abstract=2437990

3
*Unique Identification Authority of India*, Government of India, Home Page. Available at: https://uidai.gov.in

4 Initial Aadhaar privacy legislation was advanced in 2010, just a few months after Aadhaar enrollment began. *See*: [5]

5 The most recent legislation to be made public is the Privacy Bill 2014, CIS India, April 3, 2014. Available at: http://www.medianama.com/2014/04/223-leaked-privacy-bill-2014-vs-2011-cis-india/

6 Barnes [7] See page 16: “On October 19, 1904, Inspector Ferrier and Major M. W. McClaughry began fingerprinting all inmates at the Leavenworth, KS, federal prison. These fingerprint records became the beginning of the U.S. Government’s fingerprint collection.”

7
*The Integrated Automated Fingerprint Identification System* (IAFIS), Federal Bureau of Investigation. Available at: https://www.fbi.gov/services/records-management/foipa/privacy-impact-assessments/iafis

8
*Next Generation Identification officially replaces IAFIS*, CJIS Link, Volume 16, Number 2, October 2014. Available at: https://www.fbi.gov/services/cjis/cjis-link/ngi-officially-replaces-iafis-yields-more-search-options-and-investigative-leads-and-increased-identification-accuracy See also: Next Generation Identification Page, Federal Bureau of Investigation. Available at: https://www.fbi.gov/services/cjis/fingerprints-and-other-biometrics/ngi *Note*: The NGI collects multiple biometrics such as iris and fingerprint, but not DNA. DNA is collected in an FBI database called CODIS. *See*: FBI Services Page, Combined DNA Index System (CODIS). Available at: https://www.fbi.gov/services/laboratory/biometric-analysis/codis

9 See Interpol, *Databases Page*. Available at: https://www.interpol.int/INTERPOL-expertise/Databases Note: Interpol collects multiple biometrics, including DNA.

10 Manimekalai [8]. *See also*: Abdullah and Alhijily [9]. Available at: https://www.researchgate.net/publication/269987030_Biometric_in_Healthcare_Security_System_Face_-_Iris_Fusion_System Multiple vendors sell biometric technology for healthcare providers; an example of typical benefits espoused is Safran. Available at: https://usa.morpho.com/civil-identity/biometrics-healthcare

11 Solove and Hartzog [10]. 14 Bloomberg BNA Privacy & Security Law Report 1353 (2015); GWU Law School Public Law Research Paper No. 2015–33; GWU Legal Studies Research Paper No. 2015–33. Available at SSRN: https://ssrn.com/abstract=2636366

12
*About Touch ID on Apple*, Apple, Inc. Available at: https://support.apple.com/en-us/HT204587

13 Solove and Hartzog [10]. 14 Bloomberg BNA Privacy & Security Law Report 1353 (2015); GWU Law School Public Law Research Paper No. 2015–33; GWU Legal Studies Research Paper No. 2015–33. Available at SSRN: https://ssrn.com/abstract=2636366

14 Parenti [11]. See Chapter 2, Antebellum ID: Geneologies of Identification and Registration and Part 4, The Accumulation of Bodies, Part II: Early Biometrics.

15 Biometrics are the personal, physical, or behavioral characteristics of a person. This can include fingerprints, facial geometry, gait, DNA, or even ear shape. Biometrics-based information can be used to identify one specific person out of many in a one-to-many comparison, or it can be used to verify or authenticate that individual in a one-to-one comparison. Biometric systems are generally set to run in either identification or verification mode. An example of an identification system would be a law enforcement system that uses a fingerprint to search across millions of stored fingerprints for a match. An example of authentication in a one-to-one mode is that of an individual’s fingerscan to unlock their smart phone or make a mobile payment. In biometric discussions, the distinction between identification and verification is important to take into account. Formal definitions of biometrics according to ISO standards are as follows: Biometric recognition/ biometrics: “Automated recognition of individuals based on their biological and behavioural characteristics,” and biometric characteristic/ biometric (deprecated): “Biological and behavioural characteristic of an individual from which distinguishing, repeatable biometric features can be extracted for the purpose of biometric recognition.” ISO/IEC 2382–37. Information technology, Vocabulary, Part 37: Biometrics.

16 See Atick [2] Both India and Estonia have digital identity networks, among other countries. See the discussion of India’s *Aadhaar* system in this paper. See also the discussion of Estonia’s underlying data protection law in the “Policy Before Technology” section of this paper. See Also: Estonia, e-Estonia Page. Available at: https://e-estonia.com/component/electronic-id-card/

17
*World Development Report 2016: Digital Dividends*, World Bank Group. Available at: https://www.openknowledge.worldbank.org/handle/10986/23347 See also Joseph Atick, *Digital Identity: The Essential Guide*, ID4Africa Identity Forum, 2016:1–3. Available at: http://www.id4africa.com/prev/img/Digital_Identity_The_Essential_Guide.pdf

18 See Wang et al. [12]. See also Peng et al. [13]. See also Conference Papers, Biometrics Institute 2016 meeting, London: *Deep learning for face recognition: Hype or not*? Jonathon Phillips, Electronic Engineer, National Institute of Standards & Technology, USA; *Advances in 3D face recognition*, Luuk Spreeuwers, Associate Professor, Faculty for Electrical Engineering, Mathematics & Informatics, University of Twente, The Netherlands.

19 See Du and Swamy [14]

20
*Privacy by obscurity* is a term that generally refers to the inability to access especially paper-based or legacy information readily or quickly, if at all. Selinger and Hartzog [15]

21 Multimodal biometrics and biometric fusion are instances when one or more biometric attributes are used together. See an exemplar at Kaur and Neeru [16]

22
*Supra* note 20.

23 Based on Author interviews and direct observation in India, 2010–2014.

24
*Unique Identification Authority of India*, Government of India, Home Page. Available at: https://uidai.gov.in

25 Face Recognition Challenges and Evaluations, National Institute of Standards and Technology (NIST). Available at: https://www.nist.gov/programs-projects/face-projects NIST Face Recognition Challenges can be single year challenges or multi-year challenges. Challenges include: *Chexia Face Recognition* (2015–2016), *Face in Video Evaluation* (FIVE), 2014–2017; *Point and Shoot Face Recognition Challenge* (PaSC) 2015; *Face Recognition Prize Challenge* (FRPC) 2017; *Face Recognition Vendor Tests* (FRVT) 2000–2017; *Face and Ocular Challenge Series* (FOCS) 2010; *Multiple Biometric Grand Challenge* 2010; *Face Recognition Grand Challenge* (FRGC) 2010; *Face Recognition Technology* (FERET) 1993; *Text Recognition Algorithm Independent Evaluation* (TRAIT) 2015–2016.

26 See Nilekani and Shah [18]. This book was written by an individual closely associated with installing the program, as such, it is strongly biased. It nevertheless contains important documentary knowledge about how the biometric deployment took place.

27 Parliamentary Debate, *Aadhaar Act*, 2016, p. 329. Available at: http://164.100.47.132/newdebate/16/7/11032016/12To1pm.pdf

28
*Annex to the Recommendation of the Council of 23rd September 1980: Guidelines Governing The Protection of Privacy and Transborder Flows of Personal Data*, The Organization for Economic Cooperation and Development (OECD)*.* Available at: https://it.ojp.gov/documents/OECD_FIPs.pdf

29 Vaishnav [19]. *See also*: Wilson [20]. For an additional discussion of government accountability specific to India, *See also*: Mukerjee [21]

30 The *Aadhaar Act* made certain aspects of *Aadhaar* mandatory, for example, *Aadhaar* use is required for the receipt of some government services. The Aadhaar (Targeted Delivery of Financial and Other Subsidies, Benefits, and Services) Bill, 2016, Available at: http://www.prsindia.org/administrator/uploads/media/AADHAAR/Aadhaar%20Bill,%202016.pdf

31
*Aadhaar to be made mandatory for filing Income Tax return, applying for PAN Card, Times of India*, March 21, 2017. Available at: http://timesofindia.indiatimes.com/india/Aadhaar-to-be-made-mandatory-for-filing-i-t-return-applying-for-pan-card/articleshow/57756453.cms; See also Usha Ramanathan, *Blundering Along, Dangerously*, Frontline India, April 28, 2017. Available at: http://www.frontline.in/cover-story/blundering-along-dangerously/article9629188.ece?homepage=true

32 The Aadhaar card, when printed out, has 12 digits, but there are four “hidden” digits in use by the system only. For this reason, in some cases, Aadhaar is described as having 16 digits. See UID to have 12 or 16 digits? Governance Now, April 28, 2010. Available at: http://www.governancenow.com/gov-next/egov/uid-have-12-or-16-digits

33 Although it is a minor point, there have been some questions to whether *Aadhaar* issues cards. The answer is yes. But the cards are simple, and if lost, they can be reprinted from a computer. See *Aadhaar Card Information Page*. Available at: https://Aadhaarcard.in/download

34 The demographic information required for an Aadhaar number includes: Name, Date of Birth, gender, address, parent/guardian details (required for children, adults may provide), contact details phone and email (optional). The biometric information required includes: Photo, 10 finger prints, Iris scan. At this time, DNA information is not required for an *Aadhaar* card. See: Unique Identification Authority of India, FAQ on Your Aadhaar, Aadhaar Features. Available at: https://uidai.gov.in/your-aadhaar/help/faqs.html.

35 The Central Identities Repository is defined in *The Aadhaar Act of 2016* as follows: “Central Identities Data Repository” means a centralised database in one or more locations containing all *Aadhaar* numbers issued to *Aadhaar* number holders along with the corresponding demographic information and biometric information of such individuals and other information related thereto.” The Aadhaar (Targeted Delivery of Financial and Other Subsidies, Benefits and Services) Act, 2016. Available at: http://www.indiacode.nic.in/acts-in-pdf/2016/201618.pdf

36 Technical discussion of the architecture behind the *Aadhaar* identity system is readily available. Starting points include technical information from the UIDAI, UIDAI Resources Page. Available at: https://uidai.gov.in/new/resources/authentication-and-fi-documents/aadhaar-technical-documents.html Additionally, See Nilekani, Nandan and Shah, Viral. Rebooting India. Chapter 1, *Aadhaar: From Zero to One Billion in Five Years* and Chapter 5: *Going Completely Paperless with E-KYC*. See also technical illustrations in Introduction.

37 For example, in the US, the REAL ID digital system has been deeply controversial due to its potential for a centralized system. The key concerns are lack of data protections, and the ability of the government to use the proposed system in ways that could subvert existing civil liberties. See the documentation of The Identity Project, a US-based Non-Governmental Organization (NGO) for an historic timeline and documentation of REAL ID resistance. *Papers Please: REAL ID Act,* The Identity Project. Available at: https://papersplease.org/wp/category/real-id/

38 St. Hill, Aadhaar. Medium, March 22, 2017. Available at: https://medium.com/@St_Hill/i-wrote-a-few-words-about-aadhaar-34e141afb725 See also: Jharkhand's Aadhaar breach: India needs a strong data protection law. Hindustan Times, April 29, 2017. Available at: http://www.hindustantimes.com/editorials/jharkhand-s-aadhaar-breach-india-needs-a-strong-data-protection-law/story-5of3gapEnnMWiy7c7vJ4YN.html

39 While enrollments for *Aadhaar* began in late 2010, the program launched 14 months prior in 2009. Nilekani, Nandan and Shah, Viral. Rebooting India. Chapter 1, *Aadhaar: From Zero to One Billion in Five Years* and Chapter 5: *Going Completely Paperless with E-KYC*. At the time, the program was promoted as a small subsidy reform program. After two years, it was apparent that *Aadhaar* had grown in scope and it became a political struggle, however, it was too late. The politicians who wanted to pass legal protections failed in their quest, and in the vacuum of no legislation, the *Aadhaar* simply continued enrollments.

40 Available at: https://bourgeoisinspirations.files.wordpress.com/2010/03/draft_right-to-privacy.pdf

41
*Report of the Group of Experts on Privacy*, 2012. Led by Justice A.P. Shah, Former Chief Justice, High Court of Delhi, Government of India Planning Commission, October 16, 2012. Available at: http://planningcommission.nic.in/reports/genrep/rep_privacy.pdf

42
*The OECD Privacy Framework*, OECD, 2013. Available at: http://www.oecd.org/sti/ieconomy/oecd_privacy_framework.pdf See also Hoofnagle [25]

43 “Conformity with Privacy Principles: This report recommends nine fundamental Privacy Principles to form the bedrock of the proposed Privacy Act in India. These principles, drawn from best practices internationally, and adapted suitably to an Indian context, are intended to provide the baseline level of privacy protection to all individual data subjects. The fundamental philosophy underlining the principles is the need to hold the data controller accountable for the collection, processing and use to which the data is put thereby ensuring that the privacy of the data subject is guaranteed.” The principles, abridged, are: Notice, Choice and Consent, Collection Limitation, Purpose Limitation, Access and Correction, Disclosure of Information, Security, Openness, and Accountability. Excerpted from: *Report of the Group of Experts on Privacy*, 2012. Led by Justice Ajit Prakash Shah, Former Chief Justice, High Court of Delhi. Available at: http://planningcommission.nic.in/reports/genrep/rep_privacy.pdf

44 A leaked copy of the 2011 and 2014 bills is available via CIS at Leaked Privacy Bill 2014 vs. 2011, CIS India, April 3, 2014. Available at: http://www.medianama.com/2014/04/223-leaked-privacy-bill-2014-vs-2011-cis-india/

45 The Liquid Petroleum Gas (LPG) subsidy assists Indians with the purchase of fuel for cooking and heating. Available at: http://www.iisd.org/sites/default/files/publications/india_fuel_subsidies_fact_sheet.pdf

46 Here, the term *voluntariness* is used to mean “*done, made, brought about, undertaken,* etc. *of one’s own accord or by free choice*” and “*acting or done without compulsion or obligation*.” “Voluntary, voluntariness.” Dictionary.com Unabridged. Random House, Inc. 20 April 2017. Available at: http:dictionary.com/browse/voluntary

47 Despite the court order, news reports suggested that the High Court ruling was not making a difference in the “mission creep” of mandated *Aadhaar* use. Individuals holding public jobs, such as teachers, noted that they had to enroll for the *Aadhaar* or lose their position. See Early Times (India), *Despite SC decree making Aadhaar voluntary, authorities treat it compulsory*, January 14, 2016. From the article: “A Plus 2 lecturer Sumita Sharma informed Early Times that the administrative wing officials had asked her to produce the *Aadhaar* card, otherwise the salary would be withheld. ‘I had objected to the decision, giving the plea of Supreme Court judgment, but the officials simply said, your salary will not be released and it is the order of the government, after which I went for making *Aadhaar* card,’ she said.”

48
*The Aadhaar Act,* (Targeted Delivery of Financial and Other Subsidies, Benefits and Services) The Gazette of India, March 26, 2016/Chaitra 6, 1938 (Saka). Available at: http://www.prsindia.org/administrator/uploads/media/AADHAAR/Aadhaar%20Bill,%202,016.pdf

49 Parliamentary Debate 2016, 324–236 Available at: http://164.100.47.132/newdebate/16/7/11032016/12To1pm.pdf

50 See Economic Times, Budget 2016: *Full text of Finance Minister Arun Jaitley’s speech regarding The Aadhaar Act*, March 1, 2016.

51
*Unique Identification Authority of India Regulation, 2016, No. 13012/64/2016/Legal/UIDAI (No. 1 of 2016.)* the (Targeted Delivery of Financial and Other Subsidies, Benefits and Services), The Gazette of India, Sept. 12, 2016, Available at: https://github.com/cis-india/uidai-docs/blob/master/UIDAI/Act%20and%20Rules/The-Gazette-of-India_Unique-Identification-Authority-of-India-Regulations-2016_20160914.pdf For a discussion of the government of India’s procedures on access to *Aadhaar* data, see legal scholar Chinmayi Arun’s commentary, Chinmayi Arun, *Privacy is a Fundamental Right*, The Hindu, March 18, 2016, updated Sept. 6, 2016. Available at: http://www.thehindu.com/opinion/lead/lead-article-on-aadhaar-bill-by-chinmayi-arun-privacy-is-a-fundamental-right/article8366413.ece

52
*Supra* 51, Section 2(g.).

53
*Supra* note 41.

54
*The Aadhaar Act* regulates government and commercial entities. See: The Aadhaar (Targeted Delivery of Financial and Other Subsidies, Benefits, and Services) Bill, 2016, Available at: http://www.prsindia.org/administrator/uploads/media/AADHAAR/Aadhaar%20Bill,%202016.pdf The definition of government use is expansive. See **Clause 33**. Regarding external entities, see **Clause 57**. “Clause 57. -- This clause provides that nothing contained in the proposed legislation shall prevent the use of *Aadhaar* number for other purposes under law. It provides that nothing in the proposed legislation shall prevent the use of *Aadhaar* number for establishing the identity of an individual for any purpose, whether by the State or any body corporate or person, pursuant to any law, for the time being in force, or any contract to this effect, but he use of Aadhaar number under this clause shall be subject to the procedure and obligations under clause 8 and Chapter VI of the proposed legislation.”

55
*ID4D Principles on Identification*, World Bank et al., Available at: http://documents.worldbank.org/curated/en/213581486378184357/pdf/112614-REVISED-PUBLIC-web-final-ID4D-IdentificationPrinciples.pdf

56
*Aadhaar to be made mandatory for filing Income Tax return, applying for PAN Card, Times of India*, March 21, 2017. Available at: http://timesofindia.indiatimes.com/india/Aadhaar-to-be-made-mandatory-for-filing-i-t-return-applying-for-pan-card/articleshow/57756453.cms See also *Aadhaar Mandatory For Filing I-T Return, PAN Card From July 1*, CFO India, April 6, 2017. Available at: http://www.cfo-india.in/article/2017/04/06/aadhaar-mandatory-filing-i-t-return-pan-card-july-1

57 Times of India, *UGC to link scholarships to Aadhaar*, July 27, 2016. Of note, India’s Supreme Court has expressly reaffirmed that students should not be mandated to link scholarships to *Aadhaar*. However, there is not widespread compliance with the High Court stipulation. India Supreme Court of Record of Proceedings, No (s).686/2016. All Bengal Minority Students Council and ANR. VERSUS Union of India and ORS. September 14, 2016.

58 Domain-B, Railways Report: *Railways to make Aadhaar mandatory for booking of all tickets*, Sept. 13, 2016. Available at: http://www.domain-b.com/companies/companies_I/Indian_Railways/20160913_booking.html

59 MINT, *Why Aadhaar is mandatory for temple rituals at Tirupati*, July 26, 2016.

60 ESIC, *Sikkim & WB, starts linking Aadhaar number of insured persons/family members through portal,* August 11, 2016.

61 News Brief, *Newborns to get Aadhaar cards*, The Tribune India, May 1, 2015. Available at: http://www.tribuneindia.com/news/haryana/community/newborns-to-get-aadhaar-cards/74518.html

62 Editorial, *Linking midday meal to Aadhaar wrong*, Deccan Herald, March 11, 2017. Available at: http://www.deccanherald.com/content/600599/linking-midday-meal-aadhaar-wrong.html

63 Ramanathan [27] See also Ramanathan [28] The issue of conditioning rehabilitation for victims of human trafficking and prostitution upon *Aadhaar* enrollment is discussed in more depth in the *Consent* section of this paper.

64 Press Information Bureau, Government of India, *Clarification on Information Technology (Reasonable security practices and procedures and sensitive personal data or information) Rules, 2011 under section 43A of the Information Technology Act*, 2000, August 24, 2011. Available at: http://meity.gov.in/sites/upload_files/dit/files/PressNote_25811.pdf

65 Section 3 in The Information Technology (Reasonable Security Practices and Procedures and Sensitive Personal Data or Information) Rules, 2011. Available at: https://indiankanoon.org/doc/101774797/ From the Act: 3 Sensitive personal data or information.- Sensitive personal data or information of a person means such personal information which consists of information relating to: (i) password; (ii) financial information such as Bank account or credit card or debit card or other payment instrument details; (iii) physical, physiological and mental health condition; (iv) sexual orientation; (v) medical records and history; (vi) Biometric information; (vii) any detail relating to the above clauses as provided to body corporate for providing service; and (viii) any of the information received under above clauses by body corporate for processing, stored or processed under lawful contract or otherwise: Provided that, any information that is freely available or accessible in public domain or furnished under the Right to Information Act, 2005 or any other law for the time being in force shall not be regarded as sensitive personal data or information for the purposes of these rules.

66
*See*: [29].

67
*See:* [29].

68
*Supra* note 41.

69 See Eherbeck [30] *See also* World Development Report 102,725, Digital Dividends, 2016, World Bank. Available at: http://documents.worldbank.org/curated/en/896971468202972881/pdf/102725-PUB-Replacement-PUBLIC.pdf *See also* Banerjee [31]

70 The Government of India issued a report in which it acknowledged that while the speed at which the *Aadhaar* was deployed was acceptable, some States have high “failure to match” rates. The research stated that “estimates include 49% failure rates for Jharkhand, 6% for Gujarat, 5% for Krishna District in Andhra Pradesh and 37% for Rajasthan.” The report goes on to state that “Failure to identify genuine beneficiaries results in exclusion errors.” Those the *Aadhaar* intended to help are being excluded in unacceptably high numbers due to these failures. National Economic Survey, India, 2016–2017, p, 202. Available at: http://indiabudget.gov.in/es2016-17/echapter.pdf

71 National Economic Survey, India, 2016–2017: 202. Available at: http://indiabudget.gov.in/es2016-17/echapter.pdf

72 Supreme Court of India, Writ Petition (Civil) No. 494 of 2012. Available at: http://www.worldprivacyforum.org/wp-content/uploads/2015/08/SupremeCourtofIndiaAadhaar_August11_2015.pdf

73
*Supra* note 46.

74 India Supreme Court of Record of Proceedings, No(s).686/2016.*All Bengal Minority Students Council and ANR.* Versus *Union of India and ORS*. September 14, 2016. Available at: https://github.com/cis-india/uidai-docs/blob/master/Supreme%20Court%20Orders/SC_2016.09.14_Order.pdf

75
*The Aadhaar (Targeted Delivery of Financial and Other Subsidies, Benefits and Services) Act*, 2016. Available at: http://www.indiacode.nic.in/acts-in-pdf/2016/201618.pdf

76
*Supra* note 41.

77
*Aadhaar Issue*, Frontline India, April 2017. http://www.frontline.in/cover-story/database-for-a-totalitarian-state/article9629101.ece?homepage=true. *Frontline India* dedicated an issue to *Aadhaar* containing multiple articles on the topic. See also a non-partisan citizen dissent page, *Rethink Aadhaar*, launched in 2016. Available at: https://rethinkaadhaar.in

78 Sitaram Yechury, CPI(M) General Secretary and Rajya Sabha Member, as quoted in an interview in Frontline India, *Database for a totalitarian state*, April 2017. Available at: http://www.frontline.in/cover-story/database-for-a-totalitarian-state/article9629101.ece?homepage=true

79 An important example of European regulations impacting other jurisdictions is the tension between EU law and US law, which has resulted in the *EU-US Privacy Shield Agreement*. This agreement, which is complex, allows businesses to function in both jurisdictions despite differences in laws. See Robert Gellman, *US-EU Privacy Shield Analysis: Winners and Losers*, World Privacy Forum, April 6, 2016. Available at: http://www.worldprivacyforum.org/wp-content/uploads/2016/04/WPF_PrivacyShield_06April2016_fs.pdf See also Robert Gellman, *Redress Revisited: Has the Privacy Shield Agreement Between the U.S. and the EU Been Fatally Undermined by President Trump’s Executive Order 13768*? February 24, 2017. Available at: http://www.worldprivacyforum.org/wpcontent/uploads/2017/02/WPFPrivacyShield_ExecOrder_fs.pdf

80
*EU General Data Protection Regulation*, (EU-GDPR). Available at: http://www.privacy-regulation.eu/en/index.htm The GDPR will go into effect May 25, 2018. The current data protection law is EU/95/46/EC, *Directive 95/46/EC of the European Parliament and of the Council of 24 October 1995 on the protection of individuals with regard to the processing of personal data and on the free movement of such data*. Available at: http://eur-lex.europa.eu/legal-content/EN/TXT/?uri=URISERV:l14012

81 EU/95/46/EC, *Directive 95/46/EC of the European Parliament and of the Council of 24 October 1995 on the protection of individuals with regard to the processing of personal data and on the free movement of such data*. Available at: http://eur-lex.europa.eu/legal-content/EN/TXT/?uri=URISERV:l14012

82
*EU-US Privacy Shield Framework and Swiss-US Privacy Shield Framework*, US Department of Commerce. Available at: https://www.privacyshield.gov/welcome “The EU-U.S. and Swiss-U.S. Privacy Shield Frameworks were designed by the U.S. Department of Commerce and the European Commission and Swiss Administration to provide companies on both sides of the Atlantic with a mechanism to comply with data protection requirements when transferring personal data from the European Union and Switzerland to the United States in support of transatlantic commerce.”

83 See Robert Gellman, *US- EU Privacy Shield Analysis: Winners and Losers*, World Privacy Forum, April 6, 2016. Available at: http://www.worldprivacyforum.org/wp-content/uploads/2016/04/WPF_PrivacyShield_06April2016_fs.pdf

84
*EU General Data Protection Regulation*, (EU-GDPR), Definitions. Available at: http://www.privacy-regulation.eu/en/4.htm: “Consent of the data subject means any freely given, specific, and unambiguous indication of the data subject’s wishes by which he or she, by a statement or by a clear affirmative action, signifies agreement to the processing of personal data relating to him or her.”

85 See Article 6 for a complete listing. EU General Data Protection Regulation, (EU-GDPR), Article 6,"Lawfulness of processing.” Available at: http://www.privacy-regulation.eu/en/6.htm

86 For details on the EU basis of processing personal data, see *The Handbook on European Data Protection Law*, European Agency for Fundamental Human Rights, Council of Europe, 2014. Available at: http://www.echr.coe.int/Documents/Handbook_data_protection_ENG.pdf

87
*Supra* note 84.

88 Sensitive data in the EU-GDPR is defined in Article 9 of the GDPR. “Processing of personal data revealing racial or ethnic origin, political opinions, religious or philosophical beliefs, or trade union membership, and the processing of genetic data, biometric data for the purpose of uniquely identifying a natural person, data concerning health or data concerning a natural person’s sex life or sexual orientation shall be prohibited.” *EU General Data Protection Regulation*, (EU-GDPR), Article 9 “Processing of special categories of personal data.” Available at: http://www.privacy-regulation.eu/en/9.htm

89 For more on explicit Consent, see *Data Protection Directive, Art. 8 (2)* and *GDPR Article 9*. An excellent discussion of Consent in the GDPR is Gabe Maldoff, *Top 10 Operational impacts of the GDPR, Part 3, Consent*, IAPP, Jan. 12, 2016. Available at: https://iapp.org/news/a/top-10-operational-impacts-of-the-gdpr-part-3-consent/

90 Definition, “biometric data,: Article 4 EU-GDPR: “‘biometric data’ means personal data resulting from specific technical processing relating to the physical, physiological or behavioural characteristics of a natural person, which allow or confirm the unique identification of that natural person, such as facial images or dactyloscopic data.” Available at: http://www.privacy-regulation.eu/en/4.htm See also: *Supra* note 84.

91 See exemptions in EU-GDPR Article 9:2(h), (i) and 9:4. *EU General Data Protection Regulation*, (EU-GDPR), Article 9 “Processing of special categories of personal data.” Available at: http://www.privacy-regulation.eu/en/9.htm

92 See *EU General Data Protection Regulation*, (EU-GDPR), “Chapter III: Rights of the Data Subject.” Available at: http://www.privacy-regulation.eu/en/index.htm

93
*World Development Report 2016: Digital Dividends*, World Bank. Available at: https://www.openknowledge.worldbank.org/handle/10986/23347 See *Enabling Digital Development, Digital Identity*, pp. 202–197.

94 “Sector” means “A part or subdivision, especially of a society or an economy.” Harper Collins English Dictionary, “sector.” Available at: https://www.collinsdictionary.com/dictionary/english/sector Sector-based legislation is legislation that applies to just part of the economy, for example, the government sector, or the health sector.

95
*Supra* note 82*.*

96
*Supra* note 94 “Sector,” See also Paul M. Schwartz, *Preemption and Privacy*, 118 Yale L.J. 902 (2008), Available at: http://scholarship.law.berkeley.edu/facpubs/72

97 One example of this type of a gap is for individuals who are victims of medical forms of identity theft, where an individual’s identity information is used to procure health care goods or services. Victims of financial forms of identity theft can use the Federal Fair Credit Reporting Act statute to correct inaccuracies in their affected records, such as credit reports, that are caused by this crime. But victims of medical forms of ID theft do not have the commensurate right to correct their health care records under HIPAA because HIPAA does not grant patients a specific right of correcting records, even in cases of identity theft. Patients can request to add an amending statement, but they do not have the right to outright delete information from their files held by health care providers, even if inaccurate. HIPAA as a sectoral law does not include the same protections as the Fair Credit Reporting Act (which does allow for records correction), thus creating a gap in protection. See Dixon [35]

98 Health Insurance Portability and Accountability Act of 1996 (HIPAA), Public Law 104–191, 45 C.F.R. § 164.528. Available at: https://aspe.hhs.gov/report/health-insurance-portability-and-accountability-act-1996

99 Gramm-Leach-Bliley Financial Services Modernization Act (GLB Act), Title V of the Financial Services Modernization Act of 1999, Pub. L. No. 106–102, 113 Stat. 1338 (Nov. 12, 1999) (codified at 15 U.S.C. §§ 6801, 6809, 6821, and 6827) 16 C.F.R. part 313 (implementing privacy rules pursuant to GLB Act).Available at: https://www.law.cornell.edu/uscode/text/15/chapter-94/subchapter-I

100 The Fair Credit Reporting Act, 15 U.S.C. § 1681 (“FCRA”). Available at: https://www.law.cornell.edu/cfr/text/16/chapter-I/subchapter-F

101 Fair Debt Collection Practices Act (FDCPA) As amended by Public Law 111–203, title X, 124 Stat. 2092 (2010)Available at: https://www.ftc.gov/enforcement/rules/rulemaking-regulatory-reform-proceedings/fair-debt-collection-practices-act-text

102 The Family Educational Rights and Privacy Act (FERPA) (20 U.S.C. § 1232 g; 34 CFR Part 99). Available at: https://www2.ed.gov/policy/gen/guid/fpco/ferpa/index.html

103 Privacy Act of 1974, 5 U.S.C. § 552a. Available at: https://www.justice.gov/opcl/privacy-act-1974

104 See the Health State Law Database, National Conference of State Legislatures, Available at: http://www.ncsl.org/research/health/health-innovations-database.aspx This database tracks more than 800 state-level health laws that have been passed.

105
*State Identity Theft Statutes,* National Conference of State Legislatures. Available at: http://www.ncsl.org/research/financial-services-and-commerce/identity-theft-state-statutes.aspx This is a complete listing of each identity theft statute at the state level.

106 Illinois Biometric Information Privacy Act, (760 ILCS 14/) Available at: http://www.ilga.gov/legislation/ilcs/ilcs3.asp?ActID=3004&ChapterID=57 See also: The Commonwealth of Virginia has enacted the Electronic Identity Management Act (EIMA), which is not discussed in this paper as it is not a biometric or privacy regulation, but rather it is an identity management bill that focuses on establishing an identity trust framework operator, and provides limitation of liability for providers. Nevertheless, the EIMA is mentioned here as a noteworthy state-level law, as it provides the policy infrastructure for digital identity ecosystems﻿. See Commonwealth of Virginia, Electronic Identity Management Act. § 59.1-479 et seq. Available at: http://lis.virginia.gov/cgi-bin/legp604.exe?151+ful+CHAP0483

107 The Federal health privacy rule was issued by the Department of Health and Human Service under authority granted by the Health Insurance Portability and Accountability Act of 1996. The privacy rules were first issued in 2000 and became effective in 2003. There are also HIPAA rules for security. More information and copies of all the HHS rules and publications can be found at the website of the Office of Civil Rights, which is the HHS agency responsible for enforcement of the HIPAA privacy rule. Available at: http://www.hhs.gov/ocr/hipaa/

108 Confidentiality of Medical Information Act, California Civil Code Section 56–56.07 Available at: http://www.leginfo.ca.gov/cgi-bin/displaycode?section=civ&group=00001-01000&file=56-56.07

109
*Commonwealth of Virginia v. Baust*, 014–8-100. Available at: https://consumermediallc.files.wordpress.com/2014/11/245515028-fingerprint-unlock-ruling.pdf For background on this case, see Stewart [36] See also: Hulette [37]

110 The focus of this paper are the laws and policies applying *within* the legal borders of the jurisdictions discussed. In this discussion, laws regarding “border zone” uses of biometrics are not included in the analysis. Border zone biometric uses are legally complex, and require a separate and dedicated analysis. Additionally, statutes focused strictly on technical identity management frameworks have not been analyzed in this article. These kind of statutes can provide important aspects of a legal framework for digital identity ecosystems, typically apart from privacy. The EU Electronic Identification and Trust Services (eIDAS) Regulation, (Regulation 910/2014), is an example of such a statute, which provides standards for electronic transactions within the EU economic jurisdiction and regulates matters suck as electronic signatures, electronic funds transfer, electronic seals, timestamps, and other aspects of trust services. See: EUR-LEX, eIDAS, Regulation (EU) No. 910/2014. Available at: http://eur-lex.europa.eu/legal-content/EN/TXT/?uri=uriserv:OJ.L_.2014.257.01.0073.01.ENG

111 Driver’s Privacy Protection Act, Driver’s Privacy Protection Act, Pub. L. No. 103–322, Title XXX, 108 Stat. 2099 (1994). The Driver’s Privacy Protection Act prohibits the use and disclosure of some personal information (driver’s license photograph, Social Security number, driver identification number, name, address (excepting 5- digit zip code), telephone number, and medical or disability information.) that is contained in state motor vehicle records for *commercial purposes*, with some exceptions. The limitation here is that the focus of the law is for *commercial purposes* only, which limits the applicability of the law regarding government use.

112 For more about the Privacy Act see *Overview of the Privacy Act of 1974*, 2015 Ed., US Department of Justice. Available at: http://www.justice.gov/opcl/1974privacyact-overview.htm

113 The full transcripts of the HEW meetings are archived at The University of California, Berkeley. Archive of the Meetings of the Secretary’s Advisory Committee on Automated Personal Data Systems (SACAPDS), UC Berkeley. With an Introduction by Hoofnagle [39]

114 An important history of the development of Fair Information Principles is Robert Gellman, *A Basic History of Fair Information Practices*. Available at: http://bobgellman.com/rg-docs/rg-FIPShistory.pdf and http://papers.ssrn.com/sol3/papers.cfm?abstract_id=2415020

115 See Overview of the Privacy Act, US Department of Justice. Available at: https://www.justice.gov/opcl/file/793026/download This document provides analysis of key court decisions regarding the Act’s interpretation.

116
*Supra* note 42.

117
*The Federal Register.* Available at: https://www.federalregister.gov *The Federal Register* is the daily journal of the US Government and the definitive source for its official publications.

118 For a detailed discussion of the Privacy Act and law enforcement activities, see Gellman [40].

119
*The Integrated Automated Fingerprint Identification System* (IAFIS), Federal Bureau of Investigation. Available at: https://www.fbi.gov/services/records-management/foipa/privacy-impact-assessments/iafis See also IAFIS Fact Sheet, Available at: https://www.fbi.gov/file-repository/about-us-cjis-fingerprints_biometrics-biometric-center-of-excellences-iafis_0808_one-pager825/view

120 NGI Fact Page, Federal Bureau of Investigation. Available at: https://www.fbi.gov/services/cjis/fingerprints-and-other-biometrics/ngi

121 Face Recognition Technology: *DOJ and FBI Need to Take Additional Actions to Ensure Privacy and Accuracy* GAO-17-489 T: Published: Mar 22, 2017. Publicly Released: Mar 22, 2017.

122 E-Government Act of 2002, Sec. 208(b), Pub. L. No. 107–347 (Dec. 17, 2002); 44 U.S.C. 3501 note. See also M-03-22, *OMB Guidance for Implementing the Privacy Provisions of the E-Government Act of 2002* (Sept. 26, 2003).

123
*See:* [38]. For dissent regarding FBI biometric programs, see Garvie, Claire et al., *The Perpetual Lineup*, Georgetown Law Center on Privacy and Technology, Oct. 18, 2016. Available at: https://www.perpetuallineup.org/

124 REAL ID Act of 2005, P.L. 109–13. Available at: https://www.congress.gov/bill/109th-congress/house-bill/00418

125 Regarding REAL ID as a defacto national ID: Harper, Jim, REAL ID: A State-by-State Update (May 12, 2014). Cato Institute Policy Analysis No. 749. Available at SSRN: https://ssrn.com/abstract=2507479 Regarding REAL ID and its impact on immigration law: Lin, Shirley, States of Resistance: The REAL ID ACT and Constitutional Limits Upon Federal Deputization of State Agencies In The Regulation of Non-Citizens (June 1, 2009). New York City Law Review, Vol. 12, p. 329, 2009. Available at SSRN: https://ssrn.com/abstract=1550545 See also: *How the REAL ID Act is Creating a National Database,* The Identity Project. Available at: https://papersplease.org/wp/2016/02/11/how-the-real-id-act-is-creating-a-national-id-database/

126 A technical and policy discussion explaining the REAL ID Act controversy is available at How the REAL ID Act is Creating a National Database, The Identity Project. Available at: https://papersplease.org/wp/2016/02/11/how-the-real-id-act-is-creating-a-national-id-database/ See Also: US Department of Homeland Security, CFR Part 37, Minimum Standards for Driver’s Licenses and Identification Cards Acceptable by Federal Agencies for Official Purposes, Final Rule. Available at: https://www.gpo.gov/fdsys/pkg/FR-2008-01-29/pdf/08-140.pdf See also *FAQ: REAL ID, Frequently Asked Questions for the Public*, US Department of Homeland Security. Available at: https://www.dhs.gov/real-id-public-faqs. For a current list of REAL ID compliant states, see: US Department of Homeland Security, https://www.dhs.gov/current-status-states-territories

127 The Biometrics Research Group has published a report stating that biometric patient identification systems will “quickly be adopted in private clinics in the United States on a small scale. In terms of large-scale adoption, we expect that will happen internationally, as governments in emerging nations such as India and developing nations such as Ghana, adopt biometric technology to grant access to public health care programs.” Biometrics Research Group, Biometrics and Healthcare, January 2015. Available at: http://www.biometricupdate.com

128 Health records held by educational institutions do not necessarily fall under HIPAA regulations. Under the Family Educational Rights and Privacy Act (FERPA) Health records at most schools and colleges (at least those receiving federal funds) are not covered by HIPAA but by the Family Educational Rights and Privacy Act (FERPA). In general, FERPA’s protections are better than HIPAA in some ways and not as good in others. The Department of Education has published a brief guide on FERPA and HIPAA. Available at: US Department of Health and Human Services, *FAQ FERPA and HIPAA*. Available at: http://www.hhs.gov/ocr/privacy/hipaa/faq/ferpa_and_hipaa/513.html, and a more detailed guide at US Department of Education, *FERPA-HIPAA Guidance*. Available at: http://www2.ed.gov/policy/gen/guid/fpco/doc/ferpa-hipaa-guidance.pdf The interplay between HIPAA and FERPA can be complex and arcane. It is unclear how biometrics collected by health care providers covered under FERPA rather than HIPAA will ultimately operate. It is also unclear how biometrics collected by educational institutions covered under FERPA not specifically for health purposes will be handled over the long term. Few institutions are having these conversations in the public domain yet.

129
*Driver’s License Security: Federal Leadership Needed to Address Remaining Vulnerabilities*
GAO-12-893: Published: Sep 21, 2012. Publicly Released: Sep 21, 2012.

130 National Conference of State Legislatures, *Security Breach Notification Laws*, April 12, 2017. Available at: http://www.ncsl.org/research/telecommunications-and-information-technology/security-breach-notification-laws.aspx

131 Of particular note is the *Illinois Biometric Information Privacy Act*, which currently has the strongest privacy protections among US State law. The law requires entities to provide written notice to users that their biometric data is being collected, and the statute requires Consent prior to the collection of biometric data. Additional provisions relate to data retention schedule, purpose specification, and guidelines for permanent data destruction. (760 ILCS 14/) *Facebook’s Tag Suggestions Violates Illinois Biometric Privacy Law*, BNA Bloomberg, April 10, 2015. Available at: http://www.ilga.gov/legislation/ilcs/ilcs3.asp?ActID=3004&ChapterID=57 Texas: Texas BUS. & COM. CODE Ann. § 503.001

132 Biometric Information Protection Act (760 ILCS 14/) Available at: http://www.ilga.gov/legislation/ilcs/ilcs3.asp?ActID=3004&ChapterID=57

133
*Facebook’s Tag Suggestions Violates Illinois Biometric Privacy Law*, BNA Bloomberg, April 10, 2015. Available at: http://www.bna.com/suit-facebooks-tag-n17179925169/#!

134
*Consumer Privacy Bill of Rights*, The White House. Available at: https://www.whitehouse.gov/sites/default/files/privacy-final.pdf

135 “Short Form Notice Code of Conduct to Promote Transparency in Mobile App Practices” Available at: http://www.ntia.doc.gov/files/ntia/publications/july_25_code_draft.pdf

136 See generally NTIA Facial Recognition Technology MSP, Available at: http://www.ntia.doc.gov/other-publication/2013/privacy-multistakeholder-process-facial-recognition-technology

137 Center for Democracy and Technology, *CDT Withdraws from the NTIA Facial Recognition Process*, 16 June 2015. Available at: https://cdt.org/blog/cdt-withdraws-from-the-ntia-facial-recognition-process/ A joint statement came from the nine groups remaining in the process at that time.

138
*Privacy Advocates’ Statement on NTIA Facial Recognition Process*, 16 June 2015. Available at: https://www.dropbox.com/s/g7cdhl66p5um7dn/Privacy%20advocates%20statement%20on%20NTIA%20facial%20recognition%20process%20-%20FINAL.pdf?dl=0

139 Remarks of Secretary Strickland, NTIA. Available at: https://www.ntia.doc.gov/speechtestimony/2015/remarks-assistant-secretary-strickling-internet-governance-forum-usa-07162015

140 Face Recognition Technology: *DOJ and FBI Need to Take Additional Actions to Ensure Privacy and Accuracy* GAO-17-489 T: Published: Mar 22, 2017. Publicly Released: Mar 22, 2017.

141 US Federal Trade Commission, Available at: https://www.ftc.gov

142 US Department of Transportation, *Significant Rulemaking Archive 2008–2016*. Available at: https://cms.dot.gov/regulations/significant-rulemaking-report-archive

143
*Supra* note 42.

144 FIPs are the underlying regulations in numerous global regulations, including the EU, Canada, US, Australia, Japan, and others. See Greenleaf [44] See also Batch [45]. See also Kuner [46]

145 See Overview of the 2015 India High Court ruling and interim orders regarding Writ Petition (Civil) No. 494 of 2012, World Privacy Forum. Available at: http://www.hhs.gov/ohrp/regulations-and-policy/regulations/common-rule/index.html A copy of the order is available at: http://www.worldprivacyforum.org/wp-content/uploads/2015/08/SupremeCourtofIndiaAadhaar_August11_2015.pdf

146 Current Prime Minister Modi is a key moving force behind expanding *Aahhar* use. He is a proponent of linking education, medical and birth records. Nistula Hebbar, *PM Modi’s big plan: Get education, medical & birth records online in a digital locker*, Economic Times, August 29, 2014. Available at: http://articles.economictimes.indiatimes.com/2014-08-29/news/53362935_1_prime-minister-narendra-modi-suggestions-government-offices

147 For example, in August 2016, the UIDAI enabled e-Hospital services linked to *Aadhaar* numbers with enrolled mobile phones. The Maharashra State Government has directed all civic bodies to link birth certificates with *Aadhaar* cards. Civic hospitals and primary healthcare centers run by the state will implement the new rules. See also: *Pune: Aadhaar-linked birth certificates for newborns*, Express News Service, Nov. 5, 2016. See also discussion of *Aadhaar* Linked Birth Registration ALBR Project: Anjaya Anparthi, *Newborns to be enrolled under Aadhaar scheme*, Times of India, Nov. 2, 2016. See also: *To reduce organ donor fraud, organ donations to be tied to Aadhaar card: Mumbai doctors to approach state over transplant act,* Hindustan Times, Oct. 28, 2016.

148 “Women rescued from prostitution are not entitled to rehabilitation till their numbers are in the system — making anonymity the first casualty.” Usha Ramanathan, as quoted in Opinion, *A Shakey Aadhaar*, Indian Express, March 30, 2017. Available at: http://indianexpress.com/article/opinion/columns/aadhar-card-uid-supreme-court-a-shaky-aadhaar-4591671/

149 On the matter of victims being hesitant to come forward, shame and privacy are interlinked. Austin argues that shame is a marker for that which should be kept private: “Although what is private is often difficult to define, easy cases include information associated with intimacy and secrecy that lead to stigmatization and shaming if exposed.” Austin, Lisa M., Privacy, Shame and the Anxieties of Identity (January 1, 2012). Available at SSRN: https://ssrn.com/abstract=2061748 or 10.2139/ssrn.2061748

150
*Convention on Action against Trafficking in Human Beings*, The Council of Europe, Warsaw, 16.V.2005. Available at: http://www.coe.int/en/web/conventions/full-list/-/conventions/rms/090000168008371d See: “*Article 11 – Protection of private life*
1.) Each Party shall protect the private life and identity of victims. Personal data regarding them shall be stored and used in conformity with the conditions provided for by the Convention for the Protection of Individuals with regard to Automatic Processing of Personal Data (ETS No. 108). 2.) Each Party shall adopt measures to ensure, in particular, that the identity, or details allowing the identification, of a child victim of trafficking are not made publicly known, through the media or by any other means, except, in exceptional circumstances, in order to facilitate the tracing of family members or otherwise secure the well-being and protection of the child.”

151 The phrase “Orwellian” derives from the novel 1984. Orwell, George. 1984. London: Secker and Warburg, 2029. Print. Discussions of “big brother” in Orwell’s classic dystopian work refers to a controlling, pervasive government or ruling authority that takes away the privacy and civil liberties of citizens to the citizens’ detriment.

152 See discussion of Consent deemed to be freely given in EU-GDPR: (43) “In order to ensure that consent is freely given, consent should not provide a valid legal ground for the processing of personal data in a specific case where there is a clear imbalance between the data subject and the controller, in particular where the controller is a public authority and it is therefore unlikely that consent was freely given in all the circumstances of that specific situation. Consent is presumed not to be freely given if it does not allow separate consent to be given to different personal data processing operations despite it being appropriate in the individual case, or if the performance of a contract, including the provision of a service, is dependent on the consent despite such consent not being necessary for such performance.” Available at: http://data.consilium.europa.eu/doc/document/ST-5419-2016-INIT/en/pdf

153 US FDA, *A Guide to Informed Consent*. Available at: http://www.fda.gov/RegulatoryInformation/Guidances/ucm126431.htm

154 A technical and policy note here: the ability to revoke Consent can go far in introducing protections of human autonomy.⁠ Some technical aspects of this is discussed in more detail in the section on biometric encryption.

155 The European Commission has recognized Andorra, Argentina, Canada (commercial entities only), Faeroe Islands, Guernsey, Israel, Isle of Man, Jersey, New Zealand, Switzerland, and Uruguay as ensuring an adequate level of protection regarding data processing by virtue of its domestic law or international commitments. See http://ec.europa.eu/justice/data-protection/international-transfers/adequacy/index_en.htm for a roster of the decisions and opinions. Decisions regarding data transfer in the law enforcement sector are not covered by these agreements. Thus far, two stand-alone agreements have been forged in this area, the Passenger Name Record (PNR) agreement with the US, Canada, and Australia regarding air traveler identity, and the Terrorist Financing Tracking Program (TFTP) agreement with the US. Both agreements allow sharing of European data with other countries for law enforcement purposes in strictly circumscribed instances. PNR and TFTP agreements. Available at: http://ec.europa.eu/justice/data-protection/international-transfers/pnr-tftp/pnr-and-tftp_en.htm

156
*Convention for the Protection of Individuals with Regard to Automatic* *Processing of Personal Data*. Council of Europe Convention, full list. Available at: http://www.coe.int/en/web/conventions/full-list

157
*EU Charter of Fundamental Rights*, Article 8(2).

158
*Supra* note 88.

159 Article 4 of the EU-GDPR: “Consent of the data subject means any freely given, specific, and unambiguous indication of the data subject’s wishes by which he or she, by a statement or by a clear affirmative action, signifies agreement to the processing of personal data relating to him or her.” Available at: http://www.privacy-regulation.eu/en/4.htm Note that Consent in the EU can apply to processing, or to sensitive data. It is a complex topic, and this has been a brief discussion of the topic in which it is not feasible to capture all of the nuance.

160 The Article 29 Working Party is an official group in the European Union comprised of representatives from the Data Protection Authority from each EU member state, the European Data Protection Supervisor, and the European Commission. The Working Party provides expert advice to EU member states, makes recommendations to the public regarding data protection and privacy in the EU, promotes consistent application of EU data privacy law, and provides opinions to the Commission on EU laws affecting data protection and privacy. Article 29 Working Party, Opinions and Recommendations. Available at: http://ec.europa.eu/justice/data-protection/article-29/documentation/opinion-recommendation/index_en.htm

161 Article 29 Working Party Opinion 15/2011 - on the definition of Consent. Available at: http://ec.europa.eu/justice/data-protection/article-29/documentation/opinion-recommendation/files/2011/wp187_en.pdf The Working Party wrote the following about government eCards, and of Consent: “New ID cards with electronic functionalities embedded in a chip are being developed in Member States. It may not be compulsory to activate the electronic services of the card. But without activation, the user could be prevented from accessing certain administrative services, which would otherwise become difficult to reach (transfer of some services on-line, reduction of office opening hours). Consent cannot be claimed to be the legitimate ground to justify the processing. In this case the law organising the development of e-services, together with all the appropriate safeguards, should be the relevant ground.” Of note is a realistic understanding that Consent cannot be the legal basis of all privacy.

162 Definition, “biometric data,: Article 4 EU-GDPR: “‘Biometric data’ means personal data resulting from specific technical processing relating to the physical, physiological or behavioural characteristics of a natural person, which allow or confirm the unique identification of that natural person, such as facial images or dactyloscopic data.” Available at: http://www.privacy-regulation.eu/en/4.htm

163
*Supra* note 82. See also Gellman and Dixon [50]

164 Daily Mirror, *Patients the Key for US Firm*, April 28, 2016, regarding biometric distribution of iris scan technology in Northern Ireland hospitals.

165 21 CFR 50.20 General requirements for informed Consent: Except as provided in ß50.23, no investigator may involve a human being as a subject in research covered by these regulations unless the investigator has obtained the legally effective informed consent of the subject or the subject’s legally authorized representative. An investigator shall seek such Consent only under circumstances that provide the prospective subject or the representative sufficient opportunity to consider whether or not to participate and that minimize the possibility of coercion or undue influence. The information that is given to the subject or the representative shall be in language understandable to the subject or the representative. No informed Consent, whether oral or written, may include any exculpatory language through which the subject or the representative is made to waive or appear to waive any of the subject’s rights, or releases or appears to release the investigator, the sponsor, the institution, or its agents from liability for negligence. Available at: http://www.fda.gov/RegulatoryInformation/Guidances/ucm126431.htm

166 See 45 CFR part 46 and HHS, *Federal Policy for the Protections of Human Subjects* (‘Common Rule’). Available at: http://www.hhs.gov/ohrp/regulations-and-policy/regulations/common-rule/index.html

167
*Florida House Bill 1299*, introduced in 2016. The bill did not pass the responsible committee. The biometric language read: “In order to combat Medicaid fraud, by January 1, 2017, all hospitals that accept Medicaid payments must implement measures to biometrically confirm a patient’s identity.” The bill as filed, available at: https://www.flsenate.gov/Session/Bill/2016/1299/BillText/Filed/PDF

168
*The Emergency Medical Treatment and Labor Act* (EMTALA) is a US federal law with rules promulgated by the Centers for Medicare & Medicaid Serves. EMTALA is applicable to health care providers in the US that participate in Medicare and that provide emergency services. The Act requires such health care providers to provide emergency services regardless of an individual’s ability to pay. See also interpretive guidance. Available at: https://www.cms.gov/Regulations-and-Guidance/Guidance/Manuals/Downloads/som107ap_v_emerg.pdf

169 See also [54] This report defines medical identity theft, analyses the legal framework around the crime, including patient provisions for assistance, and discusses modes of the crime and potential solutions. Available at: https://www.worldprivacyforum.org/2006/05/report-medical-identity-theft-the-information-crime-that-can-kill-you/ See Right Patient, Hugh Chatham Case Study, Available at: http://www.rightpatient.com/rightpatient-hugh-chatham-case-study/ for a vendor discussion of a hospital case study in the context of fraud and biometrics. The notice of privacy practices for the case study health care provider is available at: http://www.hughchatham.org/privacy/ No mention of patient biometric identifiers is made.

170 To spoof biometric identity means the “unauthorized use of legitimate Identification & Authentication (I&A) data, however it was obtained, to mimic a subject different from the attacker.”

171 An exemplar of this can be seen in a notice provided by a US hospital based in Silicon Valley, California. Available at: https://www.elcaminohospital.org/patients-visitors-guide/admissions-registration/palmsecure

172 For a detailed history of privacy self-regulation in the US, including the original EU-US Safe Harbor Framework, see [56]

173 The World Bank Group maintains a list of all jurisdictions and the development levels of identity documents and systems. The data is available for download. See: [1]

174 [57] See: “*Article 11 – Protection of private life.*

175 An interesting example of de-linking certain aspects of politics and identity is the Estonian ID system. Estonia’s system has an “e-residency” program. This means that virtually anyone, even a non-resident citizen of another country, can acquire an Estonian e-ID. A non-resident e-ID cannot be used to vote, and there are other restrictions. The non-resident e-ID is novel in many aspects; time will tell if this, an early interoperable form of e-ID, will become global in use.

176 Estonia E-ID, e-Estonia Page. Available at: https://e-estonia.com/component/electronic-id-card/

177 See [60]

178
*Supra* note 60.

179 Privacy expert Ira Rubenstein has written a thoughtful discussion of self-regulation and analysis of alternatives. See [61]

180 The Biometrics Institute, an international expert user group, provides a forum for broad discussion. It has produced a set of broad privacy principles, which contain a discussion of the role of Consent, among other issues. The principles are available only to members. See http://www.biometricsinstitute.org/pages/privacy-charter.html and http://www.biometricsinstitute.org/pages/privacy-code.html

181
*Supra* note 42.

182 [64] See also: [65]

183
*Supra* note 66.
